# An Online Bioinformatics Curriculum

**DOI:** 10.1371/journal.pcbi.1002632

**Published:** 2012-09-13

**Authors:** David B. Searls

**Affiliations:** Independent Consultant, Philadelphia, Pennsylvania, United States of America; Whitehead Institute, United States of America

## Abstract

Online learning initiatives over the past decade have become increasingly comprehensive in their selection of courses and sophisticated in their presentation, culminating in the recent announcement of a number of consortium and startup activities that promise to make a university education on the internet, free of charge, a real possibility. At this pivotal moment it is appropriate to explore the potential for obtaining comprehensive bioinformatics training with currently existing free video resources. This article presents such a bioinformatics curriculum in the form of a virtual course catalog, together with editorial commentary, and an assessment of strengths, weaknesses, and likely future directions for open online learning in this field.

## Online Learning Comes of Age

Online academic “courseware” at the university level has now been available to the public for a decade, the earliest concerted effort having originated in 2002 with the Massachusetts Institute of Technology (MIT) and their OpenCourseWare initiative (http://ocw.mit.edu). This project offered up the syllabi, lecture notes, quizzes, exams, and/or other study materials for a very large number of courses, at the discretion of professors but with strong support and encouragement from the MIT administration. Only in a minority of cases were videos of lectures posted.

Even before this, The University of California, Berkeley, had started webcasting lectures, and eventually began posting both audio and video for public consumption at their Berkeley Webcast site (http://webcast.berkeley.edu), though without the ancillary materials of MIT's OpenCourseWare. A number of other universities followed suit, though seldom so extensively; among these was Stanford with its ClassX streaming service (http://classx.stanford.edu/ClassX) and an earlier effort called Stanford Engineering Everywhere (http://see.stanford.edu/see/courses.aspx). In many cases, individual faculty members took the initiative to post course materials, including video, in widely varying formats. Some adopted the use of “Khan-style videos” or tablet-based screencasts of the sort popularized by the Kahn Academy with its vast library of instructional videos, which started as a viral YouTube sensation and has now become its own well-funded institution (http://www.khanacademy.org).

YouTube indeed became the destination of many academic videos, which are now aggregated by institution under YouTube EDU (http://www.youtube.com/education). Apple has also put its distinctive stamp on online learning with iTunes U (http://www.apple.com/education/itunes-u), also organized by institution but with integrated search capability and, of course, deployment to iPad and iPhone apps. Countless aggregators also assemble collections of video courses, but generally with little value added.

Yale University began in 2007 to release Open Yale Courses (http://oyc.yale.edu) in a more curated and consistent format than most other efforts, including high-quality video and extensive syllabi; courses appeared incrementally, with just under 50 available to date. Then, in 2011, MIT revamped several of its online courses into a much more structured instructional format, with learning modules in outline form containing videos interspersed with self-assessment and other activities. In a somewhat different vein, the non-profit Saylor Foundation compiled a comprehensive online university curriculum comprising courses that are essentially mashups of video and text resources from many existing sources, including a number of those described above (http://www.saylor.org).

In the fall of 2011, a highly publicized online course, “Introduction to Artificial Intelligence” (AI), was conducted by Stanford University Prof. Sebastian Thrun and Google's Director of Research, Peter Norvig, based on the Stanford AI course. It ran “live” in the sense that new videos were released and homework assignments collected on a weekly basis, and quizzes and exams were given at set times, while discussion logs allowed for some degree of interaction. The course attracted 160,000 students from 190 countries, 22,000 of whom finished successfully and were granted “certificates of completion” [1]. Shortly afterwards, MIT set up a similar approach on a new platform called MITx, offering a course in electronic circuits that attracted comparable numbers of students (https://6002x.mitx.mit.edu).

The trend to structured presentation and high production quality then accelerated remarkably, and took an entrepreneurial turn. The AI course was effectively spun off by Prof. Thrun into a Web startup called Udacity (http://www.udacity.com), which is currently live with six courses. In April of 2012, two other Stanford scientists, Profs. Andrew Ng and Daphne Koller, announced a similar newco called Coursera (https://www.coursera.org), with backing from major Silicon Valley venture capital firms. Coursera, also now live, is being stocked with courses from academic partners Stanford, Princeton University, the University of Pennsylvania, and the University of Michigan; this list was recently augmented with a tranche of a dozen more top-tier universities. And in May of 2012, barely six months after MIT had rolled out its new MITx platform, they and Harvard announced that the institutions were investing $30 million each in a joint online learning initiative called edX (http://www.edxonline.org).

All of these initiatives promise to offer undiluted, highly interactive university-level courses to the public, free of charge. Moreover, there is every indication that the instruction can be effective; the U.S. Department of Education, in an exhaustive meta-analysis of 51 published head-to-head trials, found that “on average, students in online learning conditions performed better than those receiving face-to-face instruction” [2].

## An Online Bioinformatics Education

Clearly a revolution in open online learning is at hand. This is a welcome addition to a movement that also encompasses open online scientific publication, of which this journal is an example. As such, this is an appropriate forum to assess the current potential for a freely accessible online bioinformatics education.

Both the completeness and the quality of such an unconventional education should be evaluated. Such judgments cannot be entirely objective, and even curricula in conventional university settings vary widely. Thus, this must ultimately be considered an “opinion piece.” Even its purely factual content has to be viewed as evanescent, given the rate of change in online education, and the fact that newly announced initiatives may increase the selection and quality of courses available to a considerable extent even within the year.

Even so, the first opinion offered here is that it is probably already possible for a motivated student to become a competent, employable bioinformatics professional in the comfort of his or her own home—with certain important caveats to be elaborated in the discussion at the end. By way of evidence, a suggested curriculum will be laid out that is supported by existing online resources.

This central thesis, that online bioinformatics education has in some sense “arrived,” can certainly be challenged on a number of counts. The fundamental question of the optimal content for bioinformatics training would probably elude universal consensus in any case, and perhaps the most that can be hoped for is that what follows will contribute meaningfully to the dialogue. Even so, the reader has a right to question both the author's qualifications and methodology in offering these opinions.

The author has advanced degrees in both biology and computer science, has published original research in both fields, and has passing familiarity with but is by no means expert in all of the advanced course topics described below. He has helped design academic curricula as part of a major training grant and taught at both an undergraduate and graduate level, though not extensively, having spent most of his career in the computer and then the pharmaceutical industries. However, in the latter positions he was directly or indirectly responsible for hiring well over a hundred scientists and engineers for bioinformatics-related roles. Thus if any bias exists, it is probably in favor of the practical over the theoretical, though the author's own research is somewhat more in the latter category.

In terms of methodology, the author has personally sampled all of the main courses listed below that are currently available, as well as most of those offered as alternatives or suggested for advanced study. Of these, he has actually completed six of the main courses and seven in the latter categories (most recently, two of the inaugural offerings by Coursera), and has made significant progress in several more. In each case the main course offering for a given topic was adjudged superior to the alternatives based on a variety of criteria including coverage, production quality, availability of ancillary course material, and incorporation of the latest modular courseware technologies described above. Less tangible factors such as teaching style, clarity, and pace were also considered. Courses listed as alternatives to the main courses still met basic standards of quality, and in addition to offering redundancy often had other features that might appeal to specific students, for instance in terms of areas of emphasis. In several cases, courses were selected as main offerings despite being scheduled but not yet online; such judgments were made based on instructors' proven teaching backgrounds and in some instances after direct consultation with them on the syllabi.

Only courses offered without charge were considered. Online courses and entire degree programs for money are widely available, though troubling to some given issues of accreditation and mounting student debt. Course discussion logs on free resources like Coursera indicate a tremendous demand for online education in the developing world, and students anywhere may need to be thrifty, particularly if they are retraining or exploring career change. There are certainly extension programs of universities and other for-profit resources that offer good value-for-money in this arena, and those who can afford it should not be discouraged from taking advantage of such benefits as personalized instruction. Nevertheless, part of the challenge in the present instance is to see just how far the free resources have come. Moreover there is the practical issue that extending the analysis to paid courses would open up a much larger set of alternatives, most of which are inaccessible to evaluation without expenditure.

Only video courses are included, either showing the instructor with slides and/or blackboard, or in screencast format. Learning from course notes only, or even disembodied audio, simply doesn't have the immediacy of the visual experience of a lecture hall or even a tablet-based screencast. At the other extreme, one could maintain that reading textbooks at one's own speed is a more efficient and focused way to learn. That is certainly true for some, and perhaps more so for experienced and mature scholars, but it is probably also true that a lecture format offers much-needed structure to the learning process for others. Moreover, cognitive psychology offers both a theoretical basis and empirical evidence for the benefits of multimedia learning [3]. In any case, most of the courses below require reading at least selections from one or more textbooks in close coordination with the lectures (though in a surprising number of cases the textbooks are freely available online).

What follows, then, is a virtual catalog for a course of study in bioinformatics. It includes both core courses and electives, as will be evident in the commentaries included with each course. Even at that, different paths are possible depending on preparation (whether the student starts with a biology and/or computer science background already) and inclination (whether the student plans to focus on bioinformatics analysis and needs less programming experience, or hopes to develop algorithms and systems that require considerably more computational sophistication). Since this virtual program awards no degrees and makes no guarantees, it will not attempt to set absolute standards for numbers of credits and distribution of core and elective subjects, but will suggest possible study threads in the penultimate section of this article.

## Biology Department

### Fundamentals of Biology

#### Source

MIT, 7.012, Profs. Eric Lander, Robert Weinberg, Tyler Jacks, Hazel Sive, Graham Walker, Sallie Chisholm, and Dr. Michelle Mischke (Fall 2011)

#### Link

http://ocw.mit.edu/courses/biology/7-01sc-fundamentals-of-biology-fall-2011

#### Provider description

“Fundamentals of Biology focuses on the basic principles of biochemistry, molecular biology, genetics, and recombinant DNA. These principles are necessary to understanding the basic mechanisms of life and anchor the biological knowledge that is required to understand many of the challenges in everyday life, from human health and disease to loss of biodiversity and environmental quality.”

#### Commentary

Anyone motivated to enter the field of bioinformatics is unlikely to need a freshman-level introduction to biology, but this one is included for the sake of completeness. The faculty are stellar, and the course has recently been converted to modular form with interactive quizzes, problem sets, exams, and additional helpful features.

#### Alternatives

Berkeley's Biology 1A covers similar material plus somewhat more physiology and is available in several versions taught by a range of instructors, most recently one offered in Spring 2012 (http://webcast.berkeley.edu/playlist#c,d,Biology,CF8E59B3C769FB01).

#### Going further

All of the remaining courses in this virtual Department extend the material in this course in various ways.

### Principles of Evolution, Ecology, and Behavior

#### Source

Yale, EEB122, Prof. Stephen Stearns (Spring 2009)

#### Link

http://oyc.yale.edu/ecology-and-evolutionary-biology/eeb-122

#### Provider description

“This course presents the principles of evolution, ecology, and behavior for students beginning their study of biology and of the environment … Recent advances have energized these fields with results that have implications well beyond their boundaries: ideas, mechanisms, and processes that should form part of the toolkit of all biologists and educated citizens.”

#### Commentary

This is a modern treatment of evolution and ecology but not one especially geared to quantitative analysis, so may be considered optional for students of bioinformatics. Still it is a valuable reminder that molecular biology is not all there is. Especially interesting is the coverage of evolutionary medicine, in which Prof. Stearns is a leading light.

#### Alternatives

The continuation of the first-year Berkeley program, Biology 1B, spends a third of the course covering plant biology in more detail than is necessary for bioinformatics, but also provides a solid introduction to genetics and phylogeny that may be preferred as being more molecular (http://webcast.berkeley.edu/playlist#c,d,Biology,434C6A29FA3A4580). Another interesting alternative is the introductory course by Stanford Prof. Robert Sapolsky on “Human Behavioral Biology,” which actually covers a wide swath of evolution, molecular genetics, and neuroscience (http://www.youtube.com/playlist?list=PL848F2368C90DDC3D).

#### Going further

The U.S. National Institutes of Health has a series of 16 invited lectures on evolution (http://nihvideoidol1.cit.nih.gov:8080/NIH/main.jsp and click on “Lectures,” then “Evolution and Medicine”). Among a number of resources inspired by the recent Darwin centennial, one of the best is the Stanford course “Darwin's Legacy” (http://www.youtube.com/playlist?list=PLF2E17B4CDCCE15F5).

### Biochemistry

#### Source

Indian Institute of Technology (IIT), Kharagpur, BT20001, Prof. Swagata Dasgupta

#### Link

http://nptel.iitm.ac.in/video.php?subjectId=102105034

#### Provider description

“Chemistry and metabolism of biopolymers (carbohydrates, lipids, proteins, nucleic acids, and nucleoproteins), vitamins, and hormones. Amino acid, primary, secondary, tertiary, and quaternary structure of proteins … Enzymes and co-enzymes. Glycolytic pathway and TCA cycle. Electron transport and oxidative phosphorylation …”

#### Commentary

Exposure to biochemistry in greater detail than is found in the introductory biology courses is particularly recommended for those interested in biochemical pathway analysis, metabolomics, and structural bioinformatics. With this video course we introduce a resource developed by the Indian National Programme on Technology Enhanced Learning (NPTEL), whose ambition is “to build at least one version of each course offered in all of Science and Engineering in India, from BTech/BSc to PhD programs” (http://nptel.iitm.ac.in). It currently offers some 110 full video courses, skewed toward engineering, but with plans for up to 400 total. The courses tend to follow very traditional syllabi and sometimes move slowly, but are generally well produced and exhaustive in their coverage. The lectures are delivered in English that is more or less accented but nearly always impeccable, and altogether make for a rather refreshing multicultural experience.

#### Prerequisites

Introduction to Biology. Organic Chemistry.

#### Alternatives

Dr. Heather Tienson of the University of California, Los Angeles, teaches the introductory course in their biochemistry series entitled “Biochemistry: Introduction to Structure, Enzymes, and Metabolism” (http://www.oid.ucla.edu/webcasts/courses/2011-2012/2012winter/chem153a-1). The Stanford ClassX streaming service has a biochemistry course taught by Prof. Lynette Cegelski, but again it is only the first in a series of three and this one does not extend to metabolism (http://classx.stanford.edu/ClassX/system/users/web/pg/view_subject.php?subject=CHEM181_WINTER_2010_2011). Oregon State University offers a two-term course in “General Biochemistry” taught by Dr. Kevin Ahern, both of which are available, but the visuals are sometimes unclear (http://www.youtube.com/playlist?list=PL850269AA28EF394A and http://www.youtube.com/playlist?list=PL347B70A1CC0D91C6). Profs. Reginald Garrett and Charles Grisham of the University of Virginia have a free online version of their textbook “Biochemistry” [4].

### Genetics

#### Source

Berkeley, PMB 160, Profs. Robert Fischer and Jennifer Fletcher (Spring 2012)

#### Provider description

“A consideration of plant genetics and molecular biology. Principles of nuclear and organellar genome structure and function: regulation of gene expression in response to environmental and developmental stimuli; clonal analysis; investigation of the molecular and genetic bases for the exceptional cellular and developmental strategies adopted by plants.”

#### Link

http://webcast.berkeley.edu/playlist#c,d,PMB,2B7E0C3DBF1D43ED

#### Source

Berkeley, MCB C148, Profs. Daniel Barsky and Louise Glass (Spring 2011)

#### Provider description

“Course emphasizes bacterial and archaeal genetics and comparative genomics. Genetics and genomic methods used to dissect metabolic and development processes in bacteria, archaea, and selected microbial eukaryotes. Genetic mechanisms integrated with genomic information to address integration and diversity of microbial processes. Introduction to the use of computational tools for a comparative analysis of microbial genomes and determining relationships among bacteria, archaea, and microbial eukaryotes.”

#### Link

http://webcast.berkeley.edu/playlist#c,s,Spring_2011,59C08AE05E752758

#### Commentary

This pair of courses together provide in-depth coverage of classical genetics through modern genomics of the non-human variety. The first course, entitled “Plant Molecular Genetics,” actually begins with a comprehensive introduction to general Mendelian genetics, before delving into plant genetics in detail. The student may wish to skip some of the latter lectures, but they do cover many aspects of molecular genetics that are completely general. The second entry, “Microbial Genetics and Genomics,” starts halfway through the actual course with the lectures of Prof. Glass, focusing on comparative genomics, and includes an extended exercise in annotation of a new microbial genome from the Joint Genome Institute. Finally, for some exposure to current human genetics, the student should take the “Genetics for Epidemiologists” short course conducted by the National Human Genome Research Institute in 2008 (http://www.youtube.com/playlist?list=PL6D747D95EBB33F2D). While this pastiche of sources may not be ideal, it touches on the major themes in this diverse subject and will give a good sense of the tools underlying many laboratory methods used in molecular biology.

#### Prerequisites

Introduction to Biology.

#### Going further

The book “Human Molecular Genetics” by Drs. Tom Strachan and Andrew Reed, now in its 4^th^ edition, goes deeper into modern techniques [5]. Though now a bit dated, a freely accessible online version of the 2^nd^ edition is available from the National Center for Biotechnology Information (NCBI) of the U.S. National Institutes of Health (NIH) (http://www.ncbi.nlm.nih.gov/books/NBK7580).

### Molecular Biology

#### Source

Berkeley, MCB110, Profs. Thomas Alber, Qiang Zhou and Qing Zhong (Fall 2009)

#### Link

http://itunes.apple.com/WebObjects/MZStore.woa/wa/viewPodcast?id=354820440

#### Provider description

“Molecular biology of prokaryotic and eukaryotic cells and their viruses. Mechanisms of DNA replication, transcription, translation. Structure of genes and chromosomes. Regulation of gene expression. Biochemical processes and principles in membrane structure and function, intracellular trafficking and subcellular compartmentation, cytoskeletal architecture, nucleocytoplasmic transport, signal transduction mechanisms, and cell cycle control.”

#### Commentary

This upper-level Berkeley course in their Biochemistry and Molecular Biology track, which is subtitled “Macromolecular Synthesis and Cellular Function,” is a thorough introduction to basic cellular information processing and as such is important background for bioinformatics. The first third (taught by Prof. Alber) covers DNA replication and repair, the second third (Prof. Zhou) does RNA and protein synthesis, and the final third (Prof. Zhong) includes cell membranes, membrane proteins, trafficking, signaling, the cell cycle, and apoptosis. Note that there are some missing lectures in the first third of the Fall 2009 version, but the student can use the Fall 2008 version for Prof. Alber's lectures (http://itunes.apple.com/itunes-u/molecular-cell-biology-110/id354820355), which, however, is missing the final third of the course. Note that in all cases iTunes has the order of courses reversed in its listing. (An iTunes link is provided rather than a Berkeley Webcast link because a significant number of courses were dropped from the latter website during a redesign in 2011.)

#### Prerequisites

Introduction to Biology, Biochemistry, or equivalent.

### Cell and Systems Biology

#### Source

Berkeley, MCB130, Profs. Randy Schekman, Kunxin Luo and David Drubin (Spring 2009)

#### Link

http://itunes.apple.com/itunes-u/molecular-cell-biology-130/id354820424

#### Provider description

“This course is aimed at conveying an understanding of how cellular structure and function arise as a result of the properties of cellular macromolecules. An emphasis will be placed on the dynamic nature of cellular organization and will include a description of physical properties of cells (dimensions, concepts of free energy, diffusion, biophysical properties). Students will be introduced to quantitative aspects of cell biology and a view of cellular function that is based on integrating multiple pathways and modes of regulation (systems biology).”

#### Commentary

Another upper-level Berkeley course, this one in their Cell and Developmental Biology track, offers a different take on the cell that is geared to current systems biology. Berkeley does not allow this course and the previous one to be taken together for elective credit, but the overlap is mainly with the last third of the Molecular Biology course, so students may want to take only the first two thirds of that course and then this course in its entirety.

#### Prerequisites

Introduction to Biology, Biochemistry, or equivalent.

#### Going further

One particular subfield of biology that constitutes an exceedingly complex system is immunology, which has even spawned its own discipline of immunoinformatics. There are several introductory immunology courses available, including a shorter one presented from a medical perspective by Dr. Harris Goldstein of Albert Einstein Medical College (http://www.youtube.com/playlist?list=PL5703ABB5D07584D7) and another from a molecular and evolutionary standpoint by Prof. Gregory Beck of the University of Massachusetts (http://itunes.apple.com/us/itunes-u/intro-to-immunology-biol-378/id476313031).

### Eukaryotic Gene Expression

#### Source

Indian Institute of Science (IISc), Bangalore, Prof. P.N. Rangarajan

#### Link

http://nptel.iitm.ac.in/courses/104108056

#### Provider description

“[Topics include] cis-acting elements and trans-acting factors … domain structure of eukaryotic transcription factors … role of chromatin … synthesis of mRNA, rRNA, and tRNA … cell surface receptors … intracellular receptors … regulation of gene expression during development … recombinant protein expression systems … gene therapy and transgenic technology …”

#### Commentary

This NPTEL course offers a significantly more detailed view of gene regulation than the courses above, though it overlaps with them. It is not absolutely current but will still be of interest to those interested in bioinformatics of signaling pathways and genetic networks. For the larger perspective students should also view a seminar by Dr. Robert Tjian on “The Molecular Biology of Gene Regulation” (http://www.ibioseminars.org/lectures/bio-mechanisms/robert-tjian.html) and, for more recent aspects of microRNA-based regulation, talks by Dr. Adrian Ferré-D'Amaré on “Catalytic and Gene Regulatory RNAs” (http://videocast.nih.gov/launch.asp?17170), by Dr. Victor Ambros on “MicroRNA Pathways in Animal Development” (http://videocast.nih.gov/launch.asp?14844), and by Dr. Witold Filipowicz on “Regulating the Regulators: Mechanisms Controlling Function and Metabolism of microRNAs” (http://videocast.nih.gov/launch.asp?17234).

#### Prerequisites

Introduction to Biology and Biochemistry or equivalent.

### Computational Molecular Biology

#### Source

Stanford, Biochem 218, Prof. Doug Brutlag (Spring 2012)

#### Link

http://biochem218.stanford.edu

#### Provider description

“… a practical, hands-on approach to the field of computational molecular biology. The course is recommended for both molecular biologists and computer scientists desiring to understand the major issues concerning analysis of genomes, sequences and structures.”

#### Commentary

A wide-ranging bioinformatics practicum covering aspects of sequence analysis, genomics, phylogenetic reconstruction, gene regulation, and metabolic networks. There is an excellent set of slides in PDF format, which should be viewed in parallel with the video lectures, and a set of practical how-to videos as well. This course provides a biologist's approach to computational biology, and is thus listed separately from a corresponding course in the Computer Science Department. The emphasis here is more on how to use the algorithms than on the details of their construction.

#### Prerequisites

Molecular Biology.

#### Alternatives

MIT offers “Genomics and Computational Biology” by Prof. George Church (http://ocw.mit.edu/courses/health-sciences-and-technology/hst-508-genomics-and-computational-biology-fall-2002), but the online version is now 10 years old, and is audio-only so that the user must coordinate the lecture with a separate, rather massive set of slides. One hopes that the recently announced edX initiative will provide a Harvard-MIT course in this area soon. A short practical course on “DNA/Protein Sequence Analysis” is offered by Prof. Amy Denton of California State University, Channel Islands (http://itunes.apple.com/WebObjects/MZStore.woa/wa/viewPodcast?id=472584215). The author is aware of at least one graduate-level course in bioinformatics that is in preparation for one of the major online venues, but is as yet unannounced. While lacking any videos, Stanford Prof. Russ Altman's course “Representations and Algorithms for Computational Molecular Biology” has a wealth of notes, slides, readings, and other useful links (http://helix-web.stanford.edu/bmi214-2006).

#### Going further

The University of Illinois at Urbana-Champaign conducted a Summer School on “Computational Approaches for Simulation of Biological Systems” in 2003 that posted a number of videos relating to biophysical modeling and bioinformatics analyses of macromolecular structures, a topic otherwise underrepresented here (http://www.ks.uiuc.edu/Training/SumSchool/lectures.html). The laboratory of Prof. Burkhard Rost of the Technische Universität München maintains several short video courses with separate slides, having titles such as “Protein Prediction” and “Computational Systems Biology” (http://rostlab.org/cms/teaching/materials). The Canadian Bioinformatics Workshops provide a number of short courses annually on topics including pathway and network analysis, high-throughput sequencing data, metabolomics, microarrays, and cancer genomics, all of which are archived (http://bioinformatics.ca/workshops/open_access); some lecture videos are missing, but the slide sets are complete.

### Introduction to Genome Science

#### Source

University of Pennsylvania on Coursera, Profs. John Hogenesch and John Isaac Murray (Fall 2012)

#### Link

https://www.coursera.org/course/genomescience

#### Provider description

“This course serves as an introduction to the main laboratory and theoretical aspects of genomics and is divided into themes: genomes, genetics, functional genomics, systems biology, single cell approaches, proteomics, and applications. We start with the basics, DNA sequencing and the genome project, then move to high throughput sequencing methods and applications. Next we introduce principles of genetics and then apply them in clinical genetics and other large-scale sequencing projects. In the functional genomics unit, we start with RNA expression dynamics, analysis of alternative splicing, epigenomics and ChIP-seq, and metagenomics. Model organisms and forward and reverse genetics screens are then discussed, along with quantitative trait locus (QTL) and eQTL analysis. After that, we introduce integrative and single cell genomics approaches and systems biology. Finally, we conclude by introducing … proteomic approaches.”

#### Commentary

This anticipated Coursera entry promises to touch on all the “hot topics” in genomics, chip technologies, and next-generation sequencing, making it central to this curriculum. It will be closely based on the long-established core course in the Penn Graduate Group in Genomics and Computational Biology, and in fact the instructors plan to use the material with their own students. Prof. Hogenesch in particular has a strong computational orientation and indicates that the material taught in this course will be “bioinformatics-ready” (personal communication).

#### Prerequisites

Molecular Biology.

### Current Topics in Genome Analysis

#### Source

National Human Genome Research Institute (Winter 2012)

#### Link

http://www.genome.gov/12514288

#### Provider description

“A lecture series covering contemporary areas in genomics and bioinformatics.”

#### Commentary

This series of 13 extended guest lecturers in course format is offered every other year by the National Human Genome Research Institute (NHGRI) of the U.S. National Institutes of Health (NIH). Coverage includes biological sequence analysis, genome browsers, regulatory and epigenomic landscapes of mammalian genomes, next-generation sequencing technologies, population genetics, genome-wide association studies, pharmacogenomics, large-scale expression analysis, genomic medicine, and genomics of microbes and microbiomes. Handouts are provided. As part of this course, students should also do the NHGRI tutorial “Next-Gen 101” from 2011, which has 9 shorter lectures on whole-exome sequencing and analysis (http://videocast.nih.gov/launch.asp?16885), as well as the “1000 Genomes Tutorial” of 6 even shorter lectures on this important resource for bioinformatics (http://www.youtube.com/playlist?list=PLF61543E11FF78240).

#### Prerequisites

Molecular Biology.

#### Going further

The “EMBO Practical Course on Analysis of High-Throughput Sequence Data” (http://www.ebi.ac.uk/training/online/course/embo-practical-course-analysis-high-throughput-seq) is highly recommended as a hands-on introduction to modern genomic analysis. It closely coordinates video lectures with detailed analysis exercises, with tutorial handouts and code supplied, using R and Bioconductor. Topics include short read analysis, ChIP-Seq data and analysis, statistical concepts, differential expression by RNA-Seq, and allele-specific expression and eQTL.

### Biological Seminars

#### Source

Howard Hughes Medical Institute, iBioSeminars

#### Link

http://www.ibioseminars.org

#### Provider description

“iBioSeminars is a freely available library of video seminars from outstanding scientists, including many HHMI investigators. These lectures, which describe on-going research in leading laboratories, feature an extensive introduction to the subject matter, making them accessible to advanced undergraduates or beginning graduate students and researchers outside of the specific field. The main subject areas are biological mechanisms, cell biology and medicine, developmental biology and evolution, chemical biology and biophysics, and global health and energy.”

#### Commentary

Much of a biologist's advanced training is down to departmental seminars, invited speakers, conferences, etc. This star-studded collection amassed by the Howard Hughes Medical Institute now has some 80 extended seminars covering a wide range of topics, including some that are underrepresented in the available online courseware, such as neurosciences and developmental biology. An important side benefit of learning the scientific content itself is the educational experience of becoming familiar with the names, faces, and presentation techniques of many of the top scientists in the American biological community.

#### Alternatives

A particularly rich lode of talks by distinguished scientists is the NIH Director's Wednesday Afternoon Lecture series (http://videocast.nih.gov/PastEvents.asp?c=3). While there are almost 15 years' worth of these videos available for mining, the online student might be well advised to make a habit of tuning in to the live streaming of these events, for more of a flavor of the campus experience.

## Mathematics Department

### Differential Equations

#### Source

MIT, 18.03SC, Prof. Arthur Mattuck (Fall 2011)

#### Link

http://ocw.mit.edu/courses/mathematics/18-03sc-differential-equations-fall-2011

#### Provider description

“The laws of nature are expressed as differential equations. Scientists and engineers must know how to model the world in terms of differential equations, and how to solve those equations and interpret the solutions. This course focuses on the equations and techniques most useful in science and engineering.”

#### Commentary

Bioinformatics students who have somehow only studied math through integral calculus may find that some knowledge of differential equations is an important addition to their skill set. Not only are differential equations a mainstay of mathematical biology in areas such as enzyme kinetics and population dynamics, but they are the basis of many approaches to modeling of biological systems. Prof. Mattuck's development of the subject is fairly traditional, but is supplemented by updated “wrappers” in the MIT courseware that provide helpful visualizations and simulations of the sort to which many modern treatments of the subject are trending.

#### Prerequisites

Differential and Integral Calculus. Refreshers are widely available online, including vintage videos made four decades ago at MIT (when the author of this article was learning the subject there!) called “Calculus Revisited” (http://ocw.mit.edu/resources/res-18-006-calculus-revisited-single-variable-calculus-fall-2010 and http://ocw.mit.edu/resources/res-18-007-calculus-revisited-multivariable-calculus-fall-2011). The first edition of Prof. Strang's (see next course) calculus textbook is freely available online [6], as is one by U.C. San Diego's Prof. Gil Williamson [7].

#### Alternatives

The ancient MIT videos mentioned above also round out sophomore-level math with coverage of “Complex Variables, Differential Equations, and Linear Algebra” (http://ocw.mit.edu/resources/res-18-008-calculus-revisited-complex-variables-differential-equations-and-linear-algebra-fall-2011). The free online resource “Interactive Differential Equations” can be helpful for visualization (http://www.aw-bc.com/ide).

#### Going further

For classic applications of differential equations to mathematical modeling in biology, Prof. Jeffrey Chasnov of the Hong Kong University of Science and Technology makes his course notes freely available in book form (http://www.math.ust.hk/~machas/mathematical-biology.pdf). For a more recent perspective, Drs. Adam Arkin and John Doyle gave “A Short Course on Mathematical Modeling of Signaling Mechanisms in Biology” at NIH (http://videocast.nih.gov/launch.asp?9948). For those who will not be going further with formal math but would like to acquire some tools for self-defense in this arena, MIT Prof. Sanjoy Mahajan provides a free online textbook called “Street-Fighting Math” (http://ocw.mit.edu/courses/mathematics/18-098-street-fighting-mathematics-january-iap-2008/readings/sf_math.pdf).

### Numerical Methods

#### Source

University of South Florida, EML3041, Prof. Autar Kaw (Summer 2012)

#### Link

http://numericalmethods.eng.usf.edu

#### Provider description

“Numerical methods are techniques to approximate mathematical procedures … Approximations are needed because we either cannot solve the procedure analytically … or because the analytical method is intractable. In this course, you will learn the numerical methods for the following mathematical procedures and topics - Differentiation, Nonlinear Equations, Simultaneous Linear Equations, Interpolation, Regression, Integration, and Ordinary Differential Equations. Calculation of errors and their relationship to the accuracy of the numerical solutions is emphasized throughout the course.”

#### Commentary

Numerical methods are an important skill set for those who will actually need to solve differential equations and other formulations that have no easy closed form expression, which applies to a lot of real-world mathematical biology. While math packages can handle much of the dirty work, the real pros need to understand what's under the hood. While Prof. Kaw's course at the University of South Florida is listed here, this link is actually for an independent e-learning course funded by major grants to Prof. Kaw from the U.S. National Science Foundation and used by a variety of universities. It is modular, including not only hundreds of short videos but also quizzes, slides, examples, and demonstrations using a free Mathematica Player. An associated textbook is also freely available online, a chapter at a time [8]. Sample code is provided in each of Maple, MathCad, Mathematica, and MatLab, none of which are free, but the Octave free software package (http://www.gnu.org/software/octave) closely approaches the core functionality of MatLab, which is heavily used in this and several other listed courses for numerical computation and matrix math.

### Linear Algebra

#### Source

MIT, 18.06SC, Prof. Gilbert Strang (Fall 2011)

#### Link

http://ocw.mit.edu/courses/mathematics/18-06sc-linear-algebra-fall-2011

#### Provider description

“This course covers matrix theory and linear algebra, emphasizing topics useful in other disciplines such as physics, economics and social sciences, natural sciences, and engineering.”

#### Commentary

Prof. Strang is a legend as an educator, charmingly diffident in his delivery yet never lacking in clarity. He has long held that the subject of linear algebra should be given as much or more teaching emphasis than calculus and differential equations, and the rise of Big Data is now proving him correct beyond any doubt. No bioinformatics professional dealing with high-dimensional data can afford to neglect an understanding of matrix math, with many bioinformatics methods currently making use of various matrix factorizations, transformations, decompositions, and eigenwhatevers.

#### Alternatives

The charismatic Prof. N.J. Wildberger of the University of New South Wales offers a similar course (http://www.youtube.com/playlist?list=PL01A21B9E302D50C1). Prof. Jim Hefferon of Saint Michael's College has a nice introductory online textbook (http://joshua.smcvt.edu/linearalgebra).

#### Going further

The Harvard Extension School has an advanced course in “Abstract Algebra” taught by Prof. Benedict Gross, starting from a linear algebra foundation to study group theory, vector spaces, fields, etc. (http://www.extension.harvard.edu/open-learning-initiative/abstract-algebra). Prof. Edwin Connell of the University of Miami has a free online textbook “Elements of Abstract and Linear Algebra” with a similar approach (http://www.math.miami.edu/~ec/book). While these may be overkill for bioinformatics, it might just inspire some to seek deeper insights into structures in large datasets. Prof. Strang himself teaches two follow-on video courses in applied mathematics, developing his linear algebra-oriented approach to networks, structures, estimation, Fourier analysis, convolution filtering, etc. (http://ocw.mit.edu/courses/mathematics/18-085-computational-science-and-engineering-i-fall-2008 and http://ocw.mit.edu/courses/mathematics/18-086-mathematical-methods-for-engineers-ii-spring-2006). His magisterial self-published textbook for these courses includes a treatment of microarray analysis to discover “eigengenes” [9].

### Statistics

#### Source

Princeton on Coursera, Prof. Andrew Conway (Fall 2012)

#### Link

https://www.coursera.org/course/stats1

#### Provider description

“Statistics One is designed to be a friendly introduction to very simple, very basic, fundamental concepts in statistics … Random sampling and assignment. Distributions … Descriptive statistics. Measurement … Correlation. Causality … Multiple regression. Ordinary least squares … Confidence intervals. Statistical power … t-tests, chi-square tests. Analysis of Variance.”

#### Commentary

Only those with no exposure at all to statistics, or those who would benefit from a refresher, should feel the need to take this rather elementary introduction, but the skills are certainly essential to bioinformatics analysis. If necessary it can also provide a gentle lead-in to the Introduction to Probability course, which in turn will be required for more advanced work in statistics. The course makes use of the free statistical software package R (http://www.r-project.org), which bioinformatics practitioners should have in their toolbox not only for classical statistical tests taught here but for more advanced applications such as linear and nonlinear modeling, time-series analysis, classification, clustering, etc.

#### Alternatives

Udacity is offering a similar introductory course by Stanford Prof. Sebastian Thrun (http://www.udacity.com/overview/Course/st101). Profs. Susan Dean and Barbara Illowski of De Anza College offer an “Elementary Statistics” video course that also has a free online textbook and a full complement of quizzes, exams, and assignments (http://sofia.fhda.edu/gallery/statistics/index.html). For a stimulating change, one can consider learning or reviewing the basics of statistics from the perspectives of other disciplines. For instance, another way to pick up R while learning a little epidemiology is through Berkeley Prof. Tomas Aragon's course in “Applied Epidemiology using R” (http://www.youtube.com/view_play_list?p=1CBCB8C53D0CBE1F). A somewhat more detailed (but also considerably more protracted) treatment of basic research statistics is to be found in Berkeley Prof. Frederic Theunissen's “Research and Data Analysis in Psychology” (http://www.youtube.com/view_play_list?p=A07B0BAB1D82C53C). For those with more math and less time, an “Introduction to Statistical Methods for High-Energy Physics” by Prof. Glen Cowan (http://videolectures.net/cernstudentsummerschool09_cowan_is) is a four-lecture overview of material taught in the University of London course.

#### Going further

Prof. Wim Krijnen of Hanze University in the Netherlands has a free online textbook “Applied Statistics for Bioinformatics using R” [10] that does a lovely job of combining a course in statistics with instruction in R and more advanced applications to bioinformatics such as microarray analysis. Further study of statistics should be undertaken only after completing the Introduction to Probability below.

### Introduction to Probability

#### Source

Harvard, Statistics 110, Prof. Joseph Blitzstein (Fall 2011)

#### Link

http://itunes.apple.com/us/course/statistics-110-probability/id502492375

#### Provider description

“A comprehensive introduction to probability. Basics: sample spaces and events, conditional probability, and Bayes' Theorem. Univariate distributions: density functions, expectation and variance, Normal, t, Binomial, Negative Binomial, Poisson, Beta, and Gamma distributions. Multivariate distributions: joint and conditional distributions, independence, transformations, and Multivariate Normal. Limit laws: law of large numbers, central limit theorem. Markov chains: transition probabilities, stationary distributions, convergence.”

#### Commentary

Bioinformatics methods depend on statistics to a much greater degree and in much greater depth than biologists typically encounter in their training for analysis of variance and experimental design. Consequently a solid foundation in probability is de rigeur, particularly in preparation for data mining and machine learning applications. Prof. Blitzstein has an unintimidating, even laid-back style, always striving to convey valuable intuitions, but does not lack in rigor or depth of coverage.

#### Prerequisites

As noted above, those who lack even a basic working knowledge of statistics should take Statistics One, which can also serve as a less demanding lead-in to this course.

#### Alternatives

UCLA offers “Probability for Life Science” (Math 3C), a somewhat gentler approach to the topic, taught by the late Prof. Herbert Enderton (best known for his work in mathematical logic) (http://www.youtube.com/playlist?list=PL5BE09709EECF36AA&feature=plcp). The Harvard University Extension School apparently competes with the mother ship by fielding a video course by Prof. Paul Bamberg entitled “Sets, Counting, and Probability” (http://www.extension.harvard.edu/open-learning-initiative/math-sets-probability). A good textbook entitled “Introduction to Probability” by Swarthmore College Prof. Charles Grinstead and Dartmouth College Prof. J. Laurie Snell is available in a free online version [11].

#### Going further

IIT Kharagpur offers two courses through NPTEL that start with a more mathematically intensive treatment of probability founded in measure theory (usually kept “behind the curtain” for non-mathematicians), but then extend it in two different directions: “Probability and Statistics” by Prof. Somesh Kumar (http://nptel.iitm.ac.in/courses/111105041) and “Probability and Random Processes” by Prof. Mrityunjoy Chakraborty (http://www.youtube.com/playlist?list=PLD85E88483F782338). One flavor of stochastic processes that is especially important in bioinformatics is taught in “Introduction to Markov Processes” by Prof. Christof Schutte, head of the Biocomputing Group at the Freie Universität Berlin (http://www.networkmaths.ie/videos/list_videos.php?course=mar). In terms of books, a quick tour of statistical inference suited to a computer science world view can be found in the ambitiously titled “All of Statistics” by Carnegie-Mellon University Prof. Larry Wasserman [12]. For a treatment of probability, statistics, and stochastic processes that makes reference to bioinformatics throughout, see the book “Statistical Methods in Bioinformatics” by University of Pennsylvania Prof. Warren Ewens and Gregory Grant [13]. The first edition of Stanford Prof. Robert Gray's “Probability, Random Processes, and Ergodic Properties,” since reissued in a revised second edition, is freely available online [14].

### Automata

#### Source

Stanford, CS154 on Coursera, Prof. Jeffrey Ullman (Spring 2012)

#### Link

https://www.coursera.org/course/automata

#### Provider description

“The course covers four broad areas: (1) Finite automata and regular expressions, (2) Context-free grammars, (3) Turing machines and decidability, and (4) the theory of intractability, or NP-complete problems.”

#### Prerequisites

Data Structures or equivalent. Prof. Ullman recommends portions of his free online textbook “Foundations of Computer Science” as preparation [15]. The optional programming assignments require Java or Python.

#### Commentary

Despite the name, this course also extends to formal language theory and introduces tractability. The primary attraction of this Coursera offering is its illustrious instructor, who literally wrote the book on automata (and on databases, on algorithms, etc.). It's hard to imagine a better way for biologists to be introduced to the theory of computation. Topics such as automata and grammars are important in areas like pattern matching and RNA fold prediction, while an awareness of tractability and decidability is essential in contemplating algorithmic approaches to new problems. Perhaps most importantly, as Prof. Ullman points out, surveys of Stanford grads show that this course was one of the most useful in their subsequent careers, for the mindset it engendered in solving many real-world computational challenges.

#### Alternatives

For a somewhat more extensive treatment, the Harvard Extension School has an outstanding “Introduction to Formal Systems and Computation” by Prof. Harry Lewis (http://itunes.apple.com/WebObjects/MZStore.woa/wa/viewPodcast?id=429428100). A “Theory of Automata, Formal Languages and Computation” is offered by Prof. Kamala Krithivasan of IIT Madras through NPTEL, which includes lectures on natural language processing and DNA computing (http://nptel.iitm.ac.in/courses/106106049). The book by MIT Prof. Michael Sipser is standard [16], but for a free online alternative try the text by the late Prof. Eitan Gurari of Ohio State University [17].

### Discrete Math

#### Source

Stony Brook University, Prof. Steven Skiena, CSE 547 (1999)

#### Link

http://www.cs.sunysb.edu/%7Ealgorith/math-video

#### Provider description

“The mathematical analysis of algorithms uses a variety of topics from discrete mathematics—combinatorial analysis, number theory, and graph theory. The purpose of this course is to provide fluency with summations, congruences, generating functions, graph theory, and other tools of the trade. The emphasis will be on learning how to attack and solve problems.”

#### Commentary

Discrete math provides much of the theoretical foundation for computer science. At its more rarefied levels it must be considered an elective for the purposes of bioinformatics. Nevertheless it is important in the analysis of algorithms and even certain aspects of biology, and for those with ambitions to speak at RECOMB or publish in the *Journal of Computational Biology*, this sort of course is a necessary first step. The video is of marginal quality, displaying old-fashioned hand-written transparencies, so the student may wish to consider the alternatives below, but this course has the considerable advantage of closely following the truly gem-like textbook “Concrete Mathematics” by U.C. San Diego's Prof. Ronald Graham, Stanford's Prof. Donald Knuth and Oren Patashnik [18].

#### Alternatives

Udacity has an introductory course in “Logic and Discrete Mathematics” by Dr. Jonathan Farley, a mathematician (http://www.udacity.com/overview/Course/cs221). Prof. Kamala Krithivasan of IIT Madras also teaches a comprehensive math-oriented course in “Discrete Structures” via NPTEL (http://nptel.iitm.ac.in/video.php?subjectId=106106094). A textbook entitled “A Short Course in Discrete Mathematics” is now available online for free, and offers a traditional approach by U.C. San Diego Profs. Edward Bender and Gil Williamson [19].

#### Going further

Several topics that fall under the rubric of discrete math are covered more extensively by other courses in this curriculum, such as “Introduction to Probability” and “Analytic Combinatorics.” Additional topics in discrete math include Boolean algebra and mathematical logic, which are very well-covered in a Coursera offering by Stanford Prof. Michael Genesereth (https://www.coursera.org/course/intrologic).

### Analytic Combinatorics

#### Source

Princeton on Coursera, Prof. Michael Sedgewick (Spring 2013)

#### Link

https://www.coursera.org/course/introACpartI

#### Provider description

“Analytic Combinatorics aims to enable precise quantitative predictions of the properties of large combinatorial structures. The theory has emerged over recent decades as essential both for the scientific analysis of algorithms in computer science and for the study of scientific models in many other disciplines, including probability theory, statistical physics, computational biology and information theory. Part I of this course covers recurrence relations, generating functions, asymptotics, and fundamental structures such as trees, permutations, strings, tries, words, and mappings, in the context of applications to the analysis of algorithms.”

#### Commentary

Although more narrowly focused than the preceding entry, many will prefer this course as an entry point to discrete math because it will be on Coursera. It should serve as good training for the mathematical mindset and rigor of the subject area in general, and relative to some other treatments of combinatorics it will have the advantage of being closely tied to algorithms. In addition, its textbook will be made freely available online [20].

#### Alternatives

There is a free online version of another textbook by U.C. San Diego Profs. Edward Bender and Gil Williamson entitled “Foundations of Combinatorics with Applications” [21].

#### Going further

Prof. Sedgewick will also be offering Part II of this course on Coursera (https://www.coursera.org/course/introACpartII), delving further into his approach to generating functions. For a deep mathematical exploration of generating functions there is a free online version of a textbook by University of Pennsylvania Prof. Herbert Wilf, with the intriguing title “generatingfunctionology” (sic) [22].

### Networks: Theory and Application

#### Source

University of Michigan, SI 508, Prof. Lada Adamic (Winter 2009)

#### Link

http://open.umich.edu/education/si/si508/fall2008

#### Provider description

“The course covers topics in network analysis, from social networks to applications in information networks such as the Internet. I will introduce basic concepts in network theory, discuss metrics and models, use software analysis tools to experiment with a wide variety of real-world network data, and study applications to areas such as information retrieval.”

#### Commentary

Networks have a central place in current approaches to systems biology, and this course introduces important ideas about their forms and properties, using the Gephi open platform (http://gephi.org) for visualization and analysis. This course does not actually have any video in its original form, but a version of it re-labelled “Social Network Analysis” is being offered on Coursera in Fall 2012 (https://www.coursera.org/course/sna). Prof. Adamic, trained in physics like many in the field, has taught the material at both an undergraduate and graduate level, and the online version will be less demanding so as to be more generally accessible (personal communication). However, in addition to doing the online course's optional programming assignments (in R or NetLogo) the more advanced student can and should make use of the rich array of slides, tutorials, demonstrations, and sample data in the original course posting, thus using the videos as a framework to explore the course materials in greater depth. For purposes of bioinformatics, students should also take the online tutorials associated with the ubiquitous Cytoscape platform (http://www.cytoscape.org), and apply the learnings from the course itself to biological datasets wherever possible.

#### Alternatives

The wide-ranging textbook “Networks, Crowds and Markets” by Cornell Profs. David Easley and Jon Kleinberg is excellent, and a prepublication draft is available online for free [23].

#### Going further

Graph theory contributes a rich foundation of techniques to current network theory as well as underlying a large branch of the field of algorithms. Students will have some exposure to graph theory in both this course and the Algorithms course, but can find a much fuller treatment in the short course “Graph Theory and Network Analysis” taught by Prof. Paul van Dooren of the Université Catholique de Louvain (http://www.networkmaths.ie/videos/list_videos.php?course=gra), which covers not only the math (at a fairly intuitive level) but also its application to practical problems such as graph similarity, ranking, clustering, etc. Unfortunately a few lectures are missing, but all the slides are separately available (http://perso.uclouvain.be/paul.vandooren/DublinCourse.pdf). A still more comprehensive treatment of graph theory proper is offered by Prof. L. Sunil Chandran of IISc Bangalore through NPTEL (http://nptel.iitm.ac.in/courses/106108054).

### Applied Optimization

#### Source

Purdue University, Profs. Ragu Balakrishnan and Stephen Cauley (Summer 2009)

#### Link

http://www.networkmaths.ie/videos/list_videos.php?course=opt-2

#### Provider description

“1) The basic optimization problem: a) general formulation, b) special cases, c) motivating examples. 2) Linear programming: a) general form, b) Simplex method, c) applications in network flow. 3) Convex optimization: a) algorithms …, b) applications … 4) General optimization: a) mixed integer programming, b) algorithms and heuristics …”

#### Commentary

Optimization is a vast field, often associated with operations research or engineering disciplines but not seen as a core aspect of bioinformatics to date. Nevertheless applications can be found and are emerging in systems biology, modeling, experimental design, metabolic engineering, and now synthetic biology. This well-produced introductory course was actually taught by Purdue faculty in a summer program at Trinity College Dublin, the Network Mathematics Graduate Programme, along with several other courses listed in this curriculum. (Be sure to select the high resolution option on the video, and if the built-in player misbehaves simply download the MP4 files.) Students should supplement the course with the seminar “Combinatorial Optimization in Bioinformatics” by Prof. Clarisse Dhaenens of the University of Lille (http://videolectures.net/prib2010_dhaenens_oaab).

#### Prerequisites

Differential Equations, Linear Algebra.

#### Alternatives

A standard introduction to optimization is offered by Prof. Prabha Sharma of IIT Kanpur through NPTEL in “Linear Programming and Extensions” (http://nptel.iitm.ac.in/courses/111104027).

#### Going further

Stanford Engineering has two advanced courses in “Convex Optimization” by the estimable Prof. Stephen Boyd (http://see.stanford.edu/see/courseinfo.aspx?coll=2db7ced4-39d1-4fdb-90e8-364129597c87 and http://see.stanford.edu/see/courseinfo.aspx?coll=523bbab2-dcc1-4b5a-b78f-4c9dc8c7cf7a); the text book is available online for free [24]. Other free online books cover heuristic optimization methods that are of interest in bioinformatics, such as simulated annealing and genetic algorithms. One, by Prof. Sean Luke of George Mason University, offers general coverage [25], while another by Profs. Riccardo Poli and William Langdon of Essex and Prof. Nicholas McPhee of the University of Minnesota at Morris focuses on genetic algorithms [26].

### Dynamical Systems and Chaos

#### Source

Texas A&M, Math 614, Prof. Michael Pilant (2004)

#### Link

http://www.math.tamu.edu/~mpilant/math614

#### Provider description

“Discrete maps; continuous flows; dynamical systems; Poincare maps; symbolic dynamics; chaos, strange attractors; fractals; computer simulation of dynamical systems.”

#### Commentary

This should be considered an advanced elective for mathematically talented students interested in a deep understanding of dynamical systems modeling in biology. It is an individual effort by a math professor, in screencast format, with a wealth of ancillary web resources including training in MatLab. (From the main page, click “Video Lectures” on the left, and then “Archival Videos” at the top.)

#### Prerequisites

Differential Equations is essential, and Linear Algebra highly recommended.

#### Alternatives

IIT Kharagpur through NPTEL offers a course in “Chaos, Fractals, and Dynamic Systems” by Prof. Soumitro Banerjee that is similarly exhaustive but approaches the subject from an engineering perspective (http://nptel.iitm.ac.in/video.php?subjectId=108105054).

### Information Theory

#### Source

Stanford ClassX, EE376A, Prof. Tom Cover (Winter 2011)

#### Link

http://171.64.93.201/ClassX/system/users/web/pg/view_subject.php?subject=EE376A_WINTER_2010_2011

#### Provider description

“The fundamental ideas of information theory. Entropy and intrinsic randomness. Data compression to the entropy limit. Huffman coding. Arithmetic coding. Channel capacity, the communication limit. Gaussian channels. Kolmogorov complexity. Asymptotic equipartition property. Information theory and Kelly gambling. Applications to communication and data compression.”

#### Commentary

It goes without saying that much of molecular biology deals with the storage and transmission of information, which by itself makes information theory a proper topic of study for bioinformatics. Basic elements of the theory are important in machine learning approaches to data mining and appear frequently in bioinformatics tools and algorithms, including sequence motif analysis and many other applications. However, the mathematical depth of this course will only be necessary for serious theorists. The late Prof. Cover was the author of the standard textbook in the field [27].

#### Prerequisites

Introduction to Probability and general mathematical sophistication.

#### Alternatives

Stanford Prof. Tsachy Weissman will offer a new online version of this course on Coursera (http://www.infotheory-class.org). While it would be a shame to miss the chance to learn this material at the feet of the esteemed Prof. Cover, this newer version will provide the distinct benefits of a structured, modular format. The first edition of the book “Entropy and Information Theory” by Stanford Prof. Robert Gray, just reissued in a second edition, is available free online [28], as is “Information Theory, Inference, and Learning Algorithms” by Cambridge University Prof. David MacKay [29].

### Signals and Systems

#### Source

MIT, RES.6-007, Prof. Alan Oppenheim (1987)

#### Link

http://ocw.mit.edu/resources/res-6-007-signals-and-systems-spring-2011

#### Provider description

“The course presents and integrates the basic concepts for both continuous-time and discrete-time signals and systems. Signal and system representations are developed for both time and frequency domains. These representations are related through the Fourier transform and its generalizations, which are explored in detail. Filtering and filter design, modulation, and sampling for both analog and digital systems, as well as exposition and demonstration of the basic concepts of feedback systems for both analog and digital systems, are discussed and illustrated.”

#### Commentary

Traditional engineering approaches to signal processing and linear systems theory have not had a huge impact in bioinformatics to date, despite the fact that signal transduction and transmission are central aspects of cell biology. Still, some training in the engineering math that relates to feedback systems, filters, convolution, and the like is recommended as an elective, given trends in areas like systems biology and neuroinformatics. Nor should it be forgotten that Fourier analysis is at the foundation of crystallographic structure determination, and that signal processing is directly relevant to instrumentation used in omics and image processing, among other things. Although this course was recorded a quarter-century ago, it still feels very well put-together, and the eminent Prof. Oppenheim wrote the definitive text in the subject [30].

#### Prerequisites

Differential Equations. Linear Algebra and Probability are helpful. While not strictly speaking a prerequisite, the MITx course “Circuits and Electronics” introduces some of the material in Signals and Systems in a beautifully structured format, as well as teaching circuit theory that may also be very useful in studying biological networks and the neurosciences (https://6002x.mitx.mit.edu).

#### Alternatives

Prof. Mark Wickert of the University of Colorado at Colorado Springs has put up a very nice screencast series with good notes (http://www.eas.uccs.edu/wickert/ece2610). Prof. Richard Baraniuk of Rice University, who has been a long-time advocate for open source learning (http://www.ted.com/talks/richard_baraniuk_on_open_source_learning.html), maintains a free online textbook (http://cnx.org/content/col10064). In a somewhat different vein, Stanford Engineering offers an excellent course by Prof. Brad Osgood on “The Fourier Transform and its Applications” that adopts more of a deep mathematical than an engineering approach to the subject, so for those who passionately prefer “i” to “j” (and you know who you are) this may be a better choice (http://see.stanford.edu/see/courseInfo.aspx?coll=84d174c2-d74f-493d-92ae-c3f45c0ee091).

#### Going further

Prof. Wickert (see above) has also created an advanced video course on “Statistical Signal Processing” that again has good notes (http://www.eas.uccs.edu/wickert/ece5615). The book “Introduction to Statistical Signal processing” by Stanford Prof. Robert Gray and University of Maryland Prof. L. D. Davisson is freely available online [31]. For a deeper dive into modern linear systems theory, Stanford Engineering has a wonderful course by Prof. Stephen Boyd called “Introduction to Linear Dynamical Systems” (http://see.stanford.edu/see/courseinfo.aspx?coll=17005383-19c6-49ed-9497-2ba8bfcfe5f6). Linear Algebra is an absolute prerequisite for both these advanced courses, and the former would require Probability as well.

## Computer Science Department

### Introduction to Computer Science and Programming

#### Source

MIT, 6.00SC, Prof. John Guttag (Fall 2008)

#### Link

http://ocw.mit.edu/courses/electrical-engineering-and-computer-science/6-00sc-introduction-to-computer-science-and-programming-spring-2011

#### Provider description

“This subject is aimed at students with little or no programming experience. It aims to provide students with an understanding of the role computation can play in solving problems. It also aims to help students, regardless of their major, to feel justifiably confident of their ability to write small programs that allow them to accomplish useful goals. The class will use the Python programming language.”

#### Commentary

For biologists possessing only end-user experience with computers, several courses are available that offer a modest introduction to actual programming, generally in the context of an overview of computer science. This one is chosen somewhat arbitrarily, but in particular because it makes use of Python. While opinions about languages vary, a case can be made that Python is both well accepted in the bioinformatics community and pedagogically useful in encompassing many features (perhaps even too many) of object-oriented, imperative, and functional programming, in addition to ample libraries and bindings to other languages and resources. It is also a good compromise between a traditional Java or C++ language approach, which only serious bioinformatics developers will need, and the sort of lightweight scripting for string, file, and process manipulation (think Perl) that every analyst will do sooner or later. In addition, Python is simply easier to manage on a home laptop, a requirement for many online learners.

#### Alternatives

The Harvard Extension School has an “Intensive Introduction to Computer Science” that, instead of occupying a Pythonesque middle ground, uses the C language on the one hand and PHP and JavaScript on the other (http://www.extension.harvard.edu/open-learning-initiative/introduction-computer-science). MIT also offers a fascinating course that combines introductory Python programming with aspects of electrical engineering, such as signals and systems, circuits, probability and planning (http://ocw.mit.edu/courses/electrical-engineering-and-computer-science/6-01sc-introduction-to-electrical-engineering-and-computer-science-i-spring-2011). Prof. David Evans of the University of Virginia offers an “Intro to Computer Science (CS101)” on Udacity that teaches Python by building a web crawler (http://www.udacity.com/overview/Course/cs101/CourseRev/apr2012), and his textbook is also available in a free online version (though it uses Scheme rather than Python) [32]. To simply learn Python if you already have significant experience in some other language, try Nick Parlante's video tutorial at Google (https://code.google.com/edu/languages/google-python-class). He is also a long-time instructor at Stanford, and does an introductory “Computer 101” course on Coursera (https://www.coursera.org/course/cs101), yet another alternative starting point.

#### Going further

The courses above offer taster menus of various aspects of computer science and only basic programming skills, and as such are appropriate for bioinformatics professionals who need exposure to programming but will not be doing it for a living. Those who plan to do coding in-the-large or create compute-intensive applications should start with the following three courses instead, which offer greater depth and breadth in programming principles. Those who would like to focus immediately on data-driven scientific computing could do worse than “Advanced Scientific Computing with Python” taught by Berkeley Astronomy Prof. Joshua Bloom (http://itunes.apple.com/itunes-u/astronomy-250-001-spring-2012/id497766986); this course is not particularly tied to astronomy (which is wrestling with Big Data from sky surveys rather than omics), and introduces packages ranging from statistics to visualization to parallel computing, although the resolution of the videos may lead to eye strain.

### The Structure and Interpretation of Computer Programs

#### Source

Berkeley, Computer Science 61A, Prof. Paul Hilfinger (Spring 2012)

#### Link

http://webcast.berkeley.edu/playlist#c,d,Computer_Science,EE65657BC5C79469

#### Provider description

“Introduction to programming and computer science. This course exposes students to techniques of abstraction at several levels: (a) within a programming language, using higher-order functions, manifest types, data-directed programming, and message-passing; (b) between programming languages, using functional and rule-based languages as examples.”

#### Commentary

This is the first in a cycle of three core courses that Berkeley requires of computer science majors; the other two follow below. It now teaches Python 3 (after many years of using Scheme, a LISP dialect and thus more purely functional) to get across the “big ideas” of programming, covering design principles, analysis of performance, confirmation of correctness, and management of complexity. As a matter of historical interest, the title and pedigree of this course traces back to the legendary MIT course 6.001, which for decades started many computer scientists and electrical engineers on their careers, and to the associated Scheme-based book (now available online [33]) by Profs. Hal Abelson and Gerald Jay Sussman, young versions of whom can be seen delivering the full set of lectures online in 25-year-old videos (http://ocw.mit.edu/courses/electrical-engineering-and-computer-science/6-001-structure-and-interpretation-of-computer-programs-spring-2005).

#### Alternatives

Stanford Engineering has long made available its own triad of core courses for CS majors, which are only very slightly showing their age. Stanford's first course is “Programming Methodology,” which teaches Java by jumping in the deep end, paying a fair amount of attention along the way to good software engineering practice (http://see.stanford.edu/SEE/courseinfo.aspx?coll=824a47e1-135f-4508-a5aa-866adcae1111). Udacity has a post-introductory programming course taught by Google's Dr. Peter Norvig with a bit of an artificial intelligence flavor (http://www.udacity.com/overview/Course/cs212).

### Data Structures

#### Source

Berkeley, Computer Science 61B, Prof. Paul Hilfinger (Fall 2011)

#### Link

http://webcast.berkeley.edu/playlist#c,d,Computer_Science,63AE13B304CE443E

#### Provider description

“Fundamental dynamic data structures, including linear lists, queues, trees, and other linked structures; arrays, strings, and hash tables. Storage management. Elementary principles of software engineering. Abstract data types. Algorithms for sorting and searching. Introduction to the Java programming language.”

#### Commentary

Moving on from Python to Java, the Berkeley sequence not only lays out the standard toolbox of data structures but begins to sprinkle in more software engineering techniques, awareness of machine architecture, abstraction, and classic algorithms. Prof. Hilfinger, who teaches these first two courses, moves at a good clip and is unfailingly rigorous yet clear. For enterprise-wide bioinformatics programming Java is the language of choice, and the class text, “Head First Java” [34], is reputed to be one of the least painful ways to learn this (or any) language—high praise indeed.

#### Prerequisites

The Structure and Interpretation of Computer Programs or equivalent.

#### Alternatives

The second course in the Stanford sequence is “Programming Abstractions,” which covers much of the same ground as the Berkeley course but does it by introducing the C++ language (http://see.stanford.edu/SEE/courseinfo.aspx?coll=11f4f422-5670-4b4c-889c-008262e09e4e). A much more recent instantiation of this same course is currently on the Stanford ClassX streaming service (http://classx.stanford.edu/ClassX/system/users/web/pg/view_subject.php?subject=CS106B_SPRING_2010_2011).

### Machine Structures

#### Source

Berkeley, Computer Science 61C, Profs. Dan Garcia and Michael Franklin (Fall 2011)

#### Link

http://webcast.berkeley.edu/playlist#c,d,Computer_Science,B96D778365083506

#### Provider description

“The internal organization and operation of digital computers. Machine architecture, support for high-level languages (logic, arithmetic, instruction sequencing) and operating systems (I/O, interrupts, memory management, process switching). Elements of computer logic design. Tradeoffs involved in fundamental architectural design decisions.”

#### Commentary

Despite the title of this course, it brings hardware into the picture only as it relates to designing fast and memory-efficient code. The student will learn the C language, mainly because it is close to the machine, and this is still very important to bioinformatics developers who need to tune the performance of compute-intensive applications. The current version of this course touches on not only parallelism but Cloud computing, also very relevant to bioinformatics.

#### Prerequisites

Data Structures or equivalent.

#### Alternatives

The third course in the Stanford sequence is “Programming Paradigms” (http://see.stanford.edu/SEE/courseinfo.aspx?coll=2d712634-2bf1-4b55-9a3a-ca9d470755ee), which also delves into bit-level machine details and memory management using C and C++, but then also introduces the functional paradigm (with LISP) and concurrency, as well as surveying (briefly) other languages such as Python and C#. Note that the Stanford series as a whole thus teaches the languages Java, then C++, and finally a bit of LISP, Python, etc., while the Berkeley series does Python, then Java, and then C. The latter ordering is probably more appropriate for bioinformatics.

### Building Dynamic Websites

#### Source

Harvard Extension School, Computer Science E-75, Prof. David Malan (Fall 2010)

#### Link

http://cs75.tv/2010/fall

#### Provider description

“This course teaches students how to build dynamic websites with Ajax and with Linux, Apache, MySQL, and PHP (LAMP), one of today's most popular frameworks. Students learn how to set up domain names with DNS, how to structure pages with XHTML and CSS, how to program in JavaScript and PHP, how to configure Apache and MySQL, how to design and query databases with SQL, how to use Ajax with both XML and JSON, and how to build mashups. The course explores issues of security, scalability, and cross-browser support and also discusses enterprise-level deployments of websites, including third-party hosting, virtualization, colocation in data centers, firewalling, and load-balancing.”

#### Commentary

Sooner or later, anyone doing bioinformatics is likely to have to create web pages that provide data and/or services to others. Although the technologies continue to evolve rapidly, this course provides both practical experience in recent tools and good discussions of general considerations that will carry over to whatever comes down the pike next.

#### Prerequisites

Programming ability and some familiarity with HTML.

#### Alternatives

A more foundational course on “Internet Technology” is taught by Prof. Indranil Sengupta of IIT Kharagpur through NPTEL (http://nptel.iitm.ac.in/video.php?subjectId=106105084). Udacity offers a “Web Application Engineering” course taught by web entrepreneur Steve Huffman (http://www.udacity.com/overview/Course/cs253). There are a large number of practical tutorial videos available on web design and the relevant scripting languages, easily found by search.

#### Going further

One possible direction to go from here is into the realm of iPhones and iPads. Although this architecture hasn't proven friendly to bioinformatics to date, students wishing to experiment can find many online courses, including one by Stanford Prof. Paul Hegarty (http://itunes.apple.com/itunes-u/ipad-iphone-application-development/id473757255).

### Software Engineering

#### Source

Berkeley, Computer Science 169, Profs. Armando Fox and David Patterson (Spring 2012)

#### Link

http://itunes.apple.com/WebObjects/MZStore.woa/wa/viewPodcast?id=496893325

#### Provider description

“Ideas and techniques for designing, developing, and modifying large software systems. Function-oriented and object-oriented modular design techniques, designing for re-use and maintainability. Specification and documentation. Verification and validation. Cost and quality metrics and estimation. Project team organization and management.”

#### Commentary

Programming is one thing, software engineering is quite another. Bioinformatics applications are increasingly yielding to bioinformatics systems, thus the need for practitioners hoping to do significant development to study this topic in depth. The provider description is taken from the Berkeley course catalog, but in fact the instructors have lately been morphing the course toward an agile development approach to Software as a Service (SaaS) using Ruby on Rails for Cloud deployment. In other words, they are hitting many themes that are important to recent bioinformatics trends. A version of this course is also on Coursera (https://www.coursera.org/course/saas).

#### Prerequisites

Programming proficiency in an object-oriented language such as Java, C#, C++, Python, or Ruby.

#### Alternatives

MIT's approach in their “Computer System Engineering” course tends to view software and hardware as a whole, focusing on controlling complexity, strong modularity, networks, parallelism, recovery, reliability, and security (http://ocw.mit.edu/courses/electrical-engineering-and-computer-science/6-033-computer-system-engineering-spring-2009). A more traditional course, with greater emphasis on project management, is available from IIT Bombay Profs. N.L. Sarda, Umesh Bellur, and Rushikesh Joshi through NPTEL (http://nptel.iitm.ac.in/video.php?subjectId=106101061).

#### Going further

MIT also offers a higher-level course called “Performance Engineering of Software Systems” that focuses on performance analysis, algorithmic techniques for high performance, instruction-level optimizations, cache and memory hierarchy optimization, parallel programming, and building scalable distributed systems (http://ocw.mit.edu/courses/electrical-engineering-and-computer-science/6-172-performance-engineering-of-software-systems-fall-2010). A more elementary course but one that focuses on an important specific skill is Udacity's “Software Testing” by Prof. John Regehr of the University of Utah (http://www.udacity.com/overview/Course/cs258).

### Introduction to Databases

#### Source

Stanford, Prof. Jennifer Widom (Fall 2011)

#### Link

http://www.db-class.org/course

#### Provider description

“This course covers database design and the use of database management systems for applications. It includes extensive coverage of the relational model, relational algebra, and SQL. It also covers XML data including DTDs and XML Schema for validation, and the query and transformation languages XPath, XQuery, and XSLT. The course includes database design in UML, and relational design principles based on dependencies and normal forms. Many additional key database topics from the design and application-building perspective are also covered: indexes, views, transactions, authorization, integrity constraints, triggers, on-line analytical processing (OLAP), and emerging ‘NoSQL’ systems.”

#### Commentary

This is a relatively short but well-constructed course that was yet another variation on Stanford Engineering's courseware initiatives. The quizzes and short segments, presaging the approach used by Coursera, seem particularly effective for learning efficiently. This material should be considered core to bioinformatics of any stripe.

#### Alternatives

The University of Washington has an archived distance learning course by Prof. Alon Halevy (now at Google) that is titled “Introduction to Database Systems” but emphasizes data management (http://www.cs.washington.edu/education/courses/csep544/04sp). There is a more classical and in-depth database course by Profs. Dharanipragada Janakiram of IIT Madras and Srinath Srinivasa of IIT Bangalore via NPTEL (http://nptel.iitm.ac.in/video.php?subjectId=106106093).

### Computer Graphics

#### Source

UC Davis, ECS 175, Prof. Kenneth Joy (Fall 2009)

#### Link

http://itunes.apple.com/us/itunes-u/computer-graphics-fall-2009/id457893733

#### Provider description

“Principles of computer graphics. Current graphics hardware, elementary operations in two-and three-dimensional space, transformational geometry, clipping, graphics system design, standard graphics systems, individual projects.”

#### Commentary

Given the importance of scientific visualization to bioinformatics, this should be a popular elective. This course goes straight to 3D graphics, using Open GL and Qt for a considerable amount of high-level coding. It is also a good opportunity to get some exposure to graphical processing units (GPUs), which can also be used to greatly speed up non-graphical computations of relevance to bioinformatics.

#### Prerequisites

Linear Algebra, Data Structures, strong programming skills.

#### Alternatives

The Harvard Extension School has a substantially similar offering entitled “Introduction to Computer Graphics and GPU Programming” by Prof. Hanspeter Pfister and Eric Chan (http://itunes.apple.com/WebObjects/MZStore.woa/wa/viewPodcast?id=429428034). A more exhaustive introduction to the algorithms (but with no coding) is provided by IIT Madras Prof. Sukhendu Das in “Computer Graphics” via NPTEL (http://nptel.iitm.ac.in/video.php?subjectId=106106090).

#### Going further

UC Davis also offers advanced courses through their Institute for Data Analysis and Visualization, including “Graphics Architecture” (http://itunes.apple.com/us/itunes-u/graphics-architecture-winter/id404606990), which does GPUs in-depth; “Geometric Modeling” (http://itunes.apple.com/us/itunes-u/computer-science-introduction/id389259246); and “Advanced Visualization” (http://itunes.apple.com/us/itunes-u/advanced-visualization-ecs277/id389259186).

### Digital Image Processing

#### Source

Indian Institute of Technology (IIT) Kharagpur, EC61501, Prof. P.K. Biswas

#### Link

http://nptel.iitm.ac.in/video.php?subjectId=117105079

#### Provider description

“Digital image fundamentals … Image enhancement in spatial domain … Edge detection … Image filtering in frequency domain … Image restoration … Color image processing … Morphological Image Processing … Image segmentation … Texture Analysis …”

#### Commentary

Image processing has long been important in biomedical imaging and in certain omic technologies such as microarrays. It also comes into play with next-generation sequencing platforms as well as high-content screening that involves image processing of cell-based assays. This is a rigorous engineering approach to the subject for hard-core pixel jockeys.

#### Prerequisites

Differential Equations, Linear Algebra, Signals and Systems

#### Alternatives

The UC Davis program described in the previous entry also offers an “Image Processing and Analysis” course (http://itunes.apple.com/us/itunes-u/image-processing-analysis/id458753849).

#### Going further

Machine learning techniques for computer vision and image understanding are useful extensions of the basic techniques of image processing. Berkeley Prof. Jitendra Malik has a Coursera entry entitled “Computer Vision: The Fundamentals” that covers segmentation of biological images (https://www.coursera.org/course/vision). Short courses available on Videolectures.net (see Computational Seminars below) include, among others, “Learning in Computer Vision” by Prof. Simon Lucey of Carnegie Mellon University (http://videolectures.net/mlss08au_lucey_linv) and “Markov Random Fields for Vision and Graphics” by Prof. Richard Hartley of the Australian National University (http://videolectures.net/ssll09_hartley_covi). Students should first take Learning Systems or similar.

### Massively Parallel Computing

#### Source

Harvard Extension School, CSCI E-292, Profs. Hanspeter Pfister and Nicolas Pinto (Spring 2011)

#### Link

http://itunes.apple.com/WebObjects/MZStore.woa/wa/viewPodcast?id=429428651

**Provider description.** “In this course, students get hands-on experience in developing software for massively parallel computing resources. We cover parallel programming models, hardware architectures, multi-threaded programming, GPU programming, cluster computing, cloud computing, and MapReduce using Hadoop and Amazon's EC2.”

#### Commentary

Another set of skills highly relevant to current bioinformatics practice, and therefore an attractive elective. This course focuses first on GPU programming with CUDA and then on MapReduce/Hadoop programming on the Amazon Cloud. For the former, a home computer with a high-end Nvidia GPU should be sufficient (the pyCUDA Python binding is used), though online students will of course not have access to the GPU cluster used in the course. For the Cloud, EC2 accounts are free but Amazon will charge a modest amount for cycles (http://aws.amazon.com/ec2).

#### Prerequisites

Programming skills and some exposure to UNIX systems programming.

#### Alternatives

Stanford offers a course more narrowly focused on GPUs (http://itunes.apple.com/itunes-u/programming-massively-parallel/id384233322?mt=2) as well as shorter practical courses in GPUs (http://classx.stanford.edu/ClassX/system/users/web/pg/view_subject.php?subject=NVIDIA_ICME_SPRING_2010_2011), Hadoop (http://classx.stanford.edu/ClassX/system/users/web/pg/view_subject.php?subject=HADOOP_WINTER_2010_2011), and the Amazon Cloud (http://classx.stanford.edu/ClassX/system/users/web/pg/view_subject.php?subject=AEC2_WINTER_2010_2011).

### Introduction to Algorithms

#### Source

MIT, 6.046J, Profs. Charles Leiserson and Erik Demaine (Fall 2005)

#### Link

http://ocw.mit.edu/courses/electrical-engineering-and-computer-science/6-046j-introduction-to-algorithms-sma-5503-fall-2005

#### Provider description

“This course teaches techniques for the design and analysis of efficient algorithms, emphasizing methods useful in practice. Topics covered include: sorting; search trees, heaps, and hashing; divide-and-conquer; dynamic programming; amortized analysis; graph algorithms; shortest paths; network flow; computational geometry; number-theoretic algorithms; polynomial and matrix calculations; caching; and parallel computing.”

#### Commentary

The ability to understand the workings of and even create novel algorithms makes some formal training in algorithms mandatory in bioinformatics. This has never been more relevant given the volumes of data now being managed, particularly from next-generation sequencing. This is a classic course at MIT, using perhaps the most famous textbook in the field [35], co-authored by one of the instructors.

#### Prerequisites

A strong programming background. Exposure to aspects of discrete math, especially proof techniques and basic probability theory, that would be well satisfied by the Automata and Introduction to Probability courses above.

#### Alternatives

There are a number of viable alternatives. Coursera is offering a two-part course from Princeton, by Profs. Robert Sedgwick and Kevin Wayne, also using their own textbook, which however requires knowledge of Java (https://www.coursera.org/course/algs4partI). Coursera will also have the first part of the Stanford algorithms sequence, by Prof. Tim Roughgarden (https://www.coursera.org/course/algo). Berkeley offers the course taught by Profs. Christos Papadimitriou (who has bioinformatics papers as well as several textbooks among his publications) and Satish Rao (http://itunes.apple.com/itunes-u/computer-science-170-001-spring/id496893325). UC Davis has a course by Prof. Dan Gusfield, who has also published a book on computational biology algorithms [36] and includes two lectures on RNA folding in his discussion of dynamic programming (http://www.cs.ucdavis.edu/~gusfield/cs122f10/videolist.html).

#### Going further

UC Davis also offers a graduate-level algorithms course given by Prof. Gusfield (http://www.cs.ucdavis.edu/~gusfield/cs222f07/videolist.html). Meanwhile his colleague Prof. Chip Martel has his own, significantly different version of the same graduate course on iTunes U (http://itunes.apple.com/us/itunes-u/design-analysis-algorithms/id389258657).

### Computational Biology

#### Source

Stony Brook University, CSE 549, Prof. Steven Skiena (2010)

#### Link

http://www.algorithm.cs.sunysb.edu/computationalbiology

#### Provider description

“This course focuses on current problems in computational biology and bioinformatics. Our emphasis will be algorithmic, on discovering appropriate combinatorial algorithm problems and the techniques to solve them. Primary topics will include DNA sequence assembly, DNA/protein sequence assembly, DNA/protein sequence comparison, hybridization array analysis, RNA and protein folding, and phylogenic trees.”

#### Commentary

This course provides a computer scientist's approach to computational biology, and is thus listed separately from a corresponding course in the Biology Department. The emphasis here is more on how the algorithms work than on how to use them. Prof. Skiena's background is algorithms and discrete math, and he uses the book “An Introduction to Bioinformatics Algorithms” by Neil Jones and Prof. Pavel Pevzner of the University of California at San Diego [37].

#### Prerequisites

Introduction to Algorithms is recommended, though Prof. Skiena encourages the participation of biologists.

### Artificial Intelligence

#### Source

Berkeley, CS 188, Prof. Pieter Abbeel (Spring 2012)

#### Link

http://itunes.apple.com/WebObjects/MZStore.woa/wa/viewPodcast?id=496298636

#### Provider description

“Basic ideas and techniques underlying the design of intelligent computer systems. Topics include heuristic search, problem solving, game playing, knowledge representation, logical inference, planning, reasoning under uncertainty, expert systems, learning, perception, language understanding.”

#### Commentary

Bioinformatics has a long tradition relating it to artificial intelligence (AI), including the origins of the Intelligent Systems for Molecular Biology conference series. Besides introducing machine learning, which should be pursued further in the next course listed, this course introduces knowledge representation, important as a foundation for biological ontologies; Bayesian nets, useful in biological network causal analysis; and natural language understanding, which is highly relevant to biomedical text mining. The course uses Python, and refers to but does not require the very popular text by Berkeley Prof. Stuart Russell and Google's Peter Norvig, “Artificial Intelligence: A Modern Approach” [38].

#### Alternatives

As noted in the introduction, a well-publicized live course by Stanford's Prof. Sebastian Thrun and Google's Peter Norvig was offered in the Fall of 2011 (https://www.ai-class.com); the lectures and quizzes are now accessible on YouTube but in a rather awkward format. However, Prof. Thrun now has a similar AI course on Udacity, which uses Python and is keyed to programming a robotic car (http://www.udacity.com/overview/Course/cs373). The U.S. Naval Postgraduate School's Prof. Neil Rowe has a book entitled “Artificial Intelligence through Prolog” that presents some core topics using logic programming (favored by this author) and is now available online for free [39].

#### Prerequisites

Data Structures or equivalent. Basic probability and propositional logic.

#### Going further

This course provides modest coverage of the topics in the Commentary, which may well lead the interested student to pursue additional elective courses below. Students interested in robotic technologies, for instance in control of laboratory automation, should consider Stanford Prof. Oussama Khatib's course “Introduction to Robotics” (http://see.stanford.edu/see/courseinfo.aspx?coll=86cc8662-f6e4-43c3-a1be-b30d1d179743). For a look at the deepest philosophical foundations of ontologies, students may enjoy a short course by Prof. Barry Smith of the University of Buffalo entitled “An Introduction to Ontology” (http://ontology.buffalo.edu/smith/IntroOntology_Course.html). For a more computational approach, Prof. John Sowa has a well-organized but text-only “Guided Tour of Ontology” (http://www.jfsowa.com/ontology/guided.htm) that includes readings from his book “Knowledge Representation” [40]. Dr. Doug Lenat, another knowledge representation pioneer, gave an interesting seminar at NIH called “Computers versus Common Sense” (http://videocast.nih.gov/launch.asp?15085).

### Learning Systems

#### Source

California Institute of Technology, CS 156, Prof. Yaser Abu-Mostafa (Spring 2012)

#### Link

http://work.caltech.edu/telecourse.html

#### Provider description

“Introduction to the theory, algorithms, and applications of automated learning. How much information is needed to learn a task, how much computation is involved, and how it can be accomplished. Special emphasis will be given to unifying the different approaches to the subject coming from statistics, function approximation, optimization, pattern recognition, and neural networks.”

#### Commentary

Prof. Abu-Mostafa is an acclaimed teacher and the material covered is absolutely central to current bioinformatics practice. His self-published book [41] and web site are actually entitled “Learning from Data,” which gives a better sense of the relevance of the course to bioinformatics than does the Provider Description above.

#### Prerequisites

Introduction to Probability and Linear Algebra are recommended.

#### Alternatives

Stanford Engineering offers a “Machine Learning” course by Prof. Andrew Ng, also available now on Coursera (which he co-founded). It is excellent in its own way and heavily overlaps the material in this one, though with less of a data mining focus and some attention paid to robotics (http://see.stanford.edu/see/courseinfo.aspx?coll=348ca38a-3a6d-4052-937d-cb017338d7b1). There is an accompanying set of very polished course notes (http://cs229.stanford.edu/materials.html).

#### Going further

The “Machine Learning Summer School” that took place at Cambridge University in 2009 has 20 introductory and specialized tutorials of 2–3 hours each in a coordinated video and slide format (http://videolectures.net/mlss09uk_cambridge). A very popular albeit advanced text, “Elements of Statistical Learning” by Stanford Profs. Trevor Hastie, Robert Tibshirani, and Jerome Friedman, is also available online for free [42]. Students should obtain and become proficient in machine learning tools, which can be done from R or Octave (as a free alternative to MatLab) environments (see above). A friendlier user environment is provided by tools like Weka (http://www.cs.waikato.ac.nz/ml/weka), widely used in teaching, or Orange, which has add-ons for bioinformatics and text mining (http://orange.biolab.si); both are open source. Python also has resources in this arena, for example the PyML machine learning framework (http://pyml.sourceforge.net). For bioinformatics, Hidden Markov Models (HMMs) are terrifically important (not just for sequence profiles, but also Copy Number Variation discovery, Single Nucleotide Polymorphism genotyping, gene prediction, etc.). HMMs are not covered in the core courses above (though they are introduced in the course below), nor are there the same sort of user-friendly environments for HMMs, but there are toolkits that the bioinformatics student can use to study the associated algorithms, such as HMMoC (http://biowiki.org/HMMoC) or HMMConverter (http://people.cs.ubc.ca/~irmtraud/hmmconverter), as well as R packages. Stanford Prof. Daphne Koller, the other academic co-founder of Coursera, is offering a course on “Probabilistic Graphical Models,” another important flavor of machine learning that includes Bayesian nets and Markov random fields, which already have had significant impact in network bioinformatics (https://www.coursera.org/course/pgm).

### Natural Language Processing

#### Source

Stanford on Coursera, CS 224N, Profs. Dan Jurafsky and Christopher Manning (TBA)

#### Link

https://www.coursera.org/course/nlp

#### Provider description

“This course covers a broad range of topics in natural language processing, including word and sentence tokenization, text classification and sentiment analysis, spelling correction, information extraction, parsing, meaning extraction, and question answering. We will also introduce the underlying theory from probability, statistics, and machine learning that are crucial for the field, and cover fundamental algorithms like n-gram language modeling, naive bayes and maxent classifiers, sequence models like Hidden Markov Models, probabilistic dependency and constituent parsing, and vector-space models of meaning.”

#### Commentary

Not only is natural language processing (NLP) technology important in biological text mining applications, but grammars and parsing are relevant to several aspects of sequence analysis. The probabilistic methods introduced are very generally applicable to bioinformatics, especially classifiers and Hidden Markov Models. This course, or a version of it by Prof. Manning alone, is available on the Stanford Engineering open courseware site, though with some editing of videos for copyright reasons (http://see.stanford.edu/see/courseinfo.aspx?coll=63480b48-8819-4efd-8412-263f1a472f5a).

#### Prerequisites

Programming skills in Python or Java. Some Calculus, Probability, and Linear Algebra are used, but also introduced in the course. The Automata course would be excellent preparation.

#### Alternatives

A good book for self-teaching much of the basic material (also recommended by the instructor of this course, and freely available online) is “Natural Language Processing with Python” [43], which actually teaches Python alongside NLP, and introduces a powerful open source supporting library for NLP and text analytics called NLTK (Natural Language Toolkit, http://www.nltk.org).

### Computational Seminars

#### Source

Videolectures.net, Computer Science/Bioinformatics category

#### Link

http://videolectures.net/Top/Computer_Science/Bioinformatics

#### Provider description

VideoLectures.net is a free and open access educational video lectures repository. The lectures are given by distinguished scholars and scientists at the most important and prominent events like conferences, summer schools, workshops and science promotional events from many fields …”

#### Commentary

As is the case for biology, there are myriad individual seminars online in computer science. One of the best aggregations for advanced computational aspects of bioinformatics can be obtained from Videolectures.net, which consists of talks from a large number of European Union-sponsored events, many of which tend to take the form of comprehensive mini-courses. Recent meetings include ones on Machine Learning in Systems Biology, Cancer Bioinformatics, Pattern Recognition in Bioinformatics, Learning and Inference in Computational Systems Biology, and many more, amounting to a total of some 200 talks to date.

#### Alternatives

Google Tech Talks (http://www.youtube.com/user/GoogleTechTalks/videos) are another source of seminars, though with over 1,600 videos and little organization, it's necessary to use the search function judiciously. The Santa Fe Institute also has a large collection of video seminars on various aspects of complexity research, their specialty (http://santafe.edu/research/videos/catalog).

## Other Departments

### Organic Chemistry

#### Source

Yale, Chem 125A, Prof. J. Michael McBride (Fall 2008)

#### Link

http://oyc.yale.edu/chemistry/chem-125a

#### Provider description

“This is the first semester in a two-semester introductory course focused on current theories of structure and mechanism in organic chemistry, their historical development, and their basis in experimental observation. The course is open to freshmen with excellent preparation in chemistry and physics, and it aims to develop both taste for original science and intellectual skills necessary for creative research.”

#### Commentary

Computer scientists who have managed to avoid organic chemistry may benefit from the insight it provides about the molecular basis of biological systems, including the nature of the chemical bond and considerations of energy and entropy that carry over into certain computational methods. This course is especially interesting for its wide-ranging scope and historical perspective, and in particular an illuminating case study on drug testing and usage.

#### Going further

Yale also provides a second-semester continuation of this course by the same professor (http://oyc.yale.edu/chemistry/chem-125b).

#### Alternatives

UC Irvine also offers a beginning course by Prof. James Nowick, more tightly focused on straight organic chemistry (http://ocw.uci.edu/courses/Chemistry-51A-Organic-Chemistry.aspx).

### Fundamentals of Pharmacology

#### Source

University of Pennsylvania on Coursera, Prof. Emma Meagher (Summer 2012)

#### Link

https://www.coursera.org/course/pharm101

#### Provider description

“This [course] will discuss the discipline of pharmacology and its integration throughout medical science. Specifically, the content will be organized as follows: 1) Basic Pharmacological Principles; 2) Applied Pharmacology, the concept of applying the basic principles to each organ system with an emphasis on melding pathophysiology with biologic targets for drug therapy; 3) Therapeutics, considered to be the clinical application of applied pharmacology, including the financial implications of therapy, evidence-based medicine, and the limitations of drug therapy and future directions of therapeutics in all disease states, as well as the legal implications of prescription writing; and 4) Advanced Pharmacological Principles, such as cancer therapeutics.”

#### Commentary

This brief overview will be a useful elective for bioinformatics practitioners interested in drug discovery and/or translational research, from either a scientific or employment standpoint. Supplementary seminars that may be of interest include “Introduction to Drug Discovery” by Drs. James Wells and Michelle Arkin (http://www.ibioseminars.org/lectures/bio-techniques/james-wellsmichelle-arkin.html), “Imatinib (Gleevec) as a Paradigm of Targeted Cancer Therapies” by Dr. Brian Druker (http://www.ibioseminars.org/lectures/cell-bio-a-med/brian-druker.html), “Protein Kinases; Structure, Function, and Regulation” by Dr. Susan Taylor (http://www.ibioseminars.org/lectures/bio-mechanisms/susan-taylor.html), and “Seven Transmembrane Receptors” by Dr. Robert Lefkowitz (http://ibioseminars.org/lectures/cell-bio-a-med/robert-lefkowitz-1.html).

#### Going further

For an exploration of the interface of systems biology with pharmacology, the two-day NIH workshop on “Quantitative and Systems Pharmacology” held in 2008 is still very relevant (http://videocast.nih.gov/launch.asp?14673 and http://videocast.nih.gov/launch.asp?14674).

### Frontiers of Biomedical Engineering

#### Source

Yale, BENG 100, Prof. Mark Saltzman (Spring 2008)

#### Link

http://oyc.yale.edu/biomedical-engineering/beng-100

#### Provider description

“The course covers basic concepts of biomedical engineering and their connection with the spectrum of human activity. It serves as an introduction to the fundamental science and engineering on which biomedical engineering is based. Case studies of drugs and medical products illustrate the product development-product testing cycle, patent protection, and FDA approval. It is designed for science and non-science majors.”

#### Commentary

This topic falls outside the usual definition of bioinformatics, but the course has so many useful and interesting elements, including imaging, cell culture and tissue engineering, cardiovascular and renal physiology, and immunology, not to mention product development, that it is likely to make an intriguing elective.

#### Alternatives

MIT offers an “Introduction to Bioengineering,” which, however, has just a few lectures supplemented by a large number of extended “interviews” with relevant faculty (http://ocw.mit.edu/courses/biological-engineering/20-010j-introduction-to-bioengineering-be-010j-spring-2006). See also a seminar by Dr. Sangeeta Bhatia on “Tissue Engineering” (http://www.ibioseminars.org/lectures/cell-bio-a-med/sangeeta-bhatia.html).

### Game Theory

#### Source

Yale, ECON 159, Prof. Ben Polak (Fall 2007)

#### Link

http://oyc.yale.edu/economics/econ-159

#### Provider description

“This course is an introduction to game theory and strategic thinking. Ideas such as dominance, backward induction, Nash equilibrium, evolutionary stability, commitment, credibility, asymmetric information, adverse selection, and signaling are discussed and applied to games played in class and to examples drawn from economics, politics, the movies, and elsewhere.”

#### Commentary

Game theory has long been used in the study of evolutionary dynamics, an increasingly important field, and backward induction is a generalization of the same sort of dynamic programming used in biological sequence analysis, applied to such problems as choosing optimal strategies in sports. Game theory also bears on modeling and network theory. Scientists in any quantitative field probably ought to be familiar with such basic ideas as the prisoners' dilemma, Pareto optimality, and Nash equilibria, or indeed with any field that has produced eight Nobel prizes. The subject tends to be taught in economics departments, which makes for an interesting change of perspective, and also provides a link to the fascinating new field of neuroeconomics.

#### Alternatives

Stanford is planning a Coursera offering on this topic taught by an economist and computer scientist tandem, which may make it a better choice in this context (https://www.coursera.org/course/gametheory). The University of Pennsylvania's Prof. Michael Kearns mixes a little game theory and network theory together in “Networked Life,” also on Coursera (https://www.coursera.org/course/networks).

#### Going further

For an advanced, more purely mathematical approach to the subject matter, see “Non-Cooperative Game Theory” as taught by Prof. Tamer Basar of the University of Illinois at Urbana-Champaign (http://www.networkmaths.ie/videos/list_videos.php?course=game). Two worthwhile seminars relating game theory to neurosciences are “Neural Basis of Strategic Choice” by Dr. Giorgio Coricelli (http://videocast.nih.gov/launch.asp?17030) and “Neuroeconomic Approaches to Mental Disorders” by Dr. P. Read Montague (http://videocast.nih.gov/launch.asp?16632).

### Entrepreneurship

#### Source

Stanford Technology Ventures Program Entrepreneurship Corner

#### Link

http://ecorner.stanford.edu

#### Provider description

“The Stanford Technology Ventures Program (STVP) Entrepreneurship Corner is a free online archive of entrepreneurship resources for teaching and learning. The mission of the project is to support and encourage faculty around the world who teach entrepreneurship to future scientists and engineers, as well as those in management and other disciplines.”

#### Commentary

Many students who learn bioinformatics will be exposed to the very latest advances in both biotechnology and computing, probably the two fields that result in the greatest rate of business startups, especially from academic spinoffs. Thus learning entrepreneurship skills is entirely appropriate as an elective in this curriculum. The STVP is housed in Stanford Engineering and hosted by the department of Management Science and Engineering. The web site has hundreds of videos, including seminars, case studies, and tutorials, many by Silicon Valley luminaries. As a way of organizing the student's approach to this cornucopia, two collections in particular are recommended: “Invitation to Venture” (http://ecorner.stanford.edu/collections.html?collectionId=1) as an introduction, and then “Technology Ventures” (http://ecorner.stanford.edu/collections.html?collectionId=2) as a more directed approach of relevance to bioinformatics.

#### Going further

Students with strongly entrepreneurial tendencies might also wish to take a look at University of Michigan Prof. Gautam Kaul's “Introduction to Finance” on Coursera (https://www.coursera.org/course/introfinance). For the basics, there are countless economics courses online, but the Annenberg Center has a particularly nicely produced overview (http://www.learner.org/resources/series79.html).

### Justice

#### Source

Harvard, ER22, Prof. Michael Sandel (Fall 2008)

#### Link

http://www.justiceharvard.org

#### Provider description

“A critical analysis of classical and contemporary theories of justice, including discussion of present-day applications. The course examines debates about justice prominent in moral and political philosophy, and invites students to subject their own views on these controversies to critical examination.”

#### Commentary

At the inception of the Genome Project significant emphasis was placed on “ELSI” or ethical, legal, and social implications, and these are even more prominent today in such issues as personal data privacy, bioethics in human and animal experimentation, and the like. Biologists nowadays often have some training in bioethics but for computer scientists it may be more novel, yet increasingly important given new capacities for mining Big Data. This is a relatively short and very general introduction to ethics, but one that is highly intellectually stimulating—so much so that it fills a large theatre whenever it is presented at Harvard by Prof. Sandel, with production values worthy of a one-man show on Broadway. You are likely to discover useful things about yourself, for example, whether you are a deontologist or a consequentialist (which, for you computer types, has something to do with whether your moral judgments are determined at compile-time or run-time).

#### Alternatives

Oxford has a course of similar (short) length called “A Romp through Ethics for Complete Beginners,” taught by Prof. Marianne Talbot with more focus on traditional moral philosophy (http://podcasts.ox.ac.uk/series/romp-through-ethics-complete-beginners).

#### Going further

UCLA Prof. Bob Goldberg teaches an honors collegium entitled “Genetic Engineering in Medicine, Law, & Agriculture” that focuses on a range of legal and ethical issues in biotechnology (http://www.mcdb.ucla.edu/Research/Goldberg/HC70A_W12/videos.php). On Coursera from the University of Pennsylvania, Prof. Ezekiel Emanuel has a timely course on “Health Policy and the Affordable Care Act” (https://www.coursera.org/course/healthpolicy), while his colleague Prof. Jonathan Moreno will be covering the interaction of neurosciences with ethics for “Neuroethics” (https://www.coursera.org/course/neuroethics). The NIH offers a comprehensive short course on “Ethical and Regulatory Aspects of Clinical Research” (http://www.bioethics.nih.gov/hsrc and click on “Podcasts” for the videos).

## Courses of Study

As noted at the outset, students will come to online learning from different backgrounds and with different goals in mind, and moreover will have different amounts of time to devote to the process. Therefore it is not helpful to be overly prescriptive about course selection. However, it is possible to identify some basic “types” of bioinformatics practitioners, and to suggest possible course selections best suited to those career paths. It should be emphasized that different institutions and individuals may have other views on bioinformatics curricula, disagreeing on appropriate electives and even on core courses. To this, the author can only plead editorial privilege, and remind the reader that these are opinions based on one person's experience in the field. It would be prudent for potential students to seek a variety of opinions.

### Curriculum Tracks

We identify below a set of five possible tracks, noting two-letter abbreviations used in Tables 1–4 where the recommended distributions of courses for each track are indicated using symbols defined in the key at the bottom of each table. (See individual course descriptions above for explanations of source abbreviations and further elaboration of requirements.) There may well be other paths, and certainly a variety of more specialized ones, but these broad categories would seem to be a useful start.

**Table 1:** Biology Department curriculum with recommended tracks.

Course	Source	BA	DM	BT	SW	CB
Fundamentals of Biology	MIT	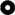	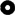	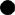	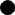	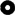
Principles of Evolution, Ecology, & Behavior	Yale	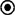	○			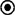
Biochemistry	NPTEL	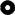	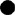	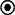	○	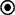
Genetics	Berkeley	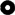	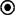	○	○	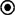
Molecular Biology	Berkeley	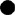	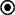	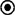	○	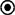
Cell and Systems Biology	Berkeley	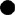	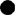	○		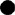
Eukaryotic Gene Expression	NPTEL	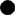	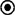	○		○
Introduction to Genome Science	U. Penn	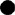	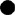	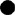	○	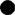
Computational Molecular Biology	Stanford	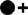	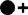	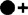	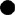	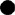
Current Topics in Genome Analysis	NHGRI	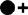	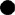	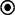	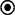	○
Biological Seminars	HHMI	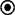	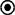	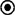	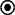	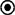

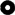
: Prerequisite; 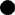: Core; 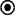: Recommended; ○: Elective; **+**: Independent Study.

**Table 2:** Mathematics Department curriculum with recommended tracks.

Course	Source	BA	DM	BT	SW	CB
Differential Equations	MIT	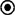	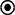			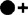
Numerical Methods	U. S. Florida		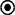	○		○
Linear Algebra	MIT	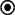	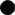	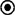	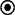	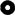
Statistics	Princeton	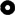	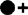	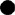	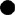	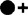
Introduction to Probability	Harvard	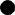	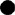	& 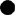	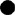	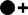
Automata	Stanford		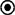	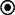	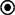	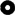
Discrete Math	Stony Brook		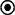	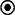	○	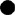
Analytic Combinatorics	Princeton		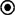	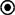	○	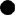
Networks: Theory and Application	U. Michigan	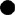	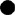	○	○	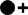
Applied Optimization	Purdue		○	○		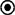
Dynamical Systems and Chaos	Texas A&M		○			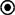
Information Theory	Stanford		○			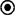
Signals and Systems	MIT		○		○	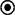

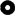
: Prerequisite; 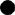: Core; 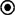: Recommended; ○: Elective; **+**: Independent Study.

**Table 3:** Computer Science Department curriculum with recommended tracks.

Course	Source	BA	DM	BT	SW	CB
Introduction to Computer Science & Programming	MIT	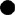				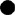
Structure & Interpretation of Computer Programs	Berkeley	○	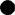	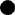	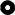	○
Data Structures	Berkeley		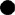	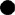	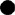	
Machine Structures	Berkeley		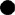	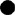	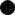	
Building Dynamic Websites	Harvard	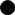	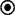	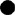	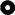	○
Software Engineering	Berkeley			○	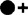	
Introduction to Databases	Stanford	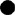	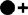	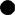	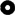	○
Computer Graphics	UC Davis	○	○	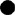	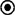	○
Digital Image Processing	NPTEL		○	○	○	
Massively Parallel Computing	Harvard		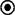	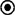	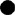	○
Introduction to Algorithms	MIT	○	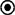	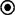	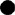	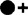
Computational Biology	Stony Brook	○	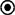	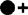	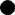	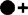
Artificial Intelligence	Berkeley	○	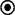	○	○	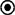
Learning Systems	Cal Tech	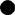	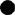	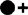	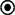	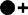
Natural Language Processing	Stanford	○	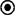	○	○	○
Computational Seminars	E.U.		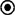	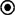	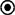	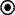

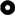
: Prerequisite; 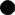: Core; 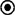: Recommended; ○: Elective; **+**: Independent Study.

**Table 4:** Other Departments curriculum with recommended tracks.

Course	Source	BA	DM	BT	SW	CB
Organic Chemistry	Yale	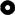	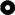	○	○	○
Fundamentals of Pharmacology	U Penn	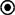	○	○	○	○
Frontiers of Biomedical Engineering	Yale	○	○	○	○	○
Game Theory	Yale	○	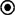	○	○	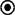
Entrepreneurship	Stanford	○	○	○	○	○
Justice	Harvard	○	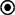	○	○	○

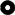
: Prerequisite; 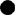: Core; 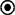: Recommended; ○: Elective; **+**: Independent Study.

In Tables 1–4, the courses in each virtual department indicated as prerequisites for a given track represent an assumed background for individuals entering the track, and should certainly be taken if the material is unfamiliar or needs refreshing. Core courses are those deemed central to the track, and should be taken if the material has not already been mastered elsewhere. Electives are at the option of the student, but certain of these are indicated as recommended, and several at least should be taken as time permits. Finally, for some tracks, additional study is recommended to extend certain course topics (denoted by plus signs), as discussed below under Independent Study.

#### Bioinformatics Analysis (BA)

This track prepares an individual to do biological data analysis with a view to interpretation or prediction. It involves such skills as sequence, expression, and functional analysis by means of a standard bioinformatics tool set, as well as an ability to write computational scripts, database queries, and simple programs.

#### Data Mining (DM)

This track begins with the analyst skill set but goes further to enable more sophisticated analyses of datasets that are especially complex, for example, by virtue of being very large scale, noisy, high-dimensional, semantically rich, poorly organized or integrated, etc. It entails a greater depth of both mathematical knowledge and programming skills.

#### Bioinformatics Tools (BT)

This track is meant to afford the capability to develop standalone tools of significant sophistication for bioinformatics analysis, visualization, presentation, and local data management. It requires programming skills in a variety of languages and the ability to implement complex algorithms efficiently, based on solid biological domain knowledge.

#### Bioinformatics Systems (BS)

This track adds to the previous one the competency for software engineering in-the-large, at a level sufficient to participate in or lead the development of major bioinformatics systems and/or products, for instance supporting data management and analysis from novel technological platforms through complex downstream analysis pipelines.

#### Computational Biology (CB)

This track is intended to prepare individuals to do original research in biological modeling and analysis by way of advanced mathematical and computational techniques. It provides a deeper grounding in computer science and engineering disciplines relevant to the sciences of complexity, information, and systems.

### Independent Study

Even in a university environment, it is not unusual for the classes that are necessary or desirable for a given course of study to be unavailable when needed. Certainly the curriculum above is constrained by the available online courses, as discussed below in the conclusion. In addition the patchwork nature of the courses, arising as they do from many institutions, can be a strength but also a weakness, with less opportunity for coordination and seamless sequencing of course contents. As in academia, any gaps can be addressed, or special interests accommodated, by independent study. The major disadvantage is the lack of a faculty mentor, which requires students to be proactive, self-sufficient, and conscientious in discerning the needs and means for supplementing their coursework. Perhaps the best way to approach this is for students to make a habit of reading the key journals in their field so as to discover systematic gaps in their knowledge.

The type of independent study needed will depend on the background of the student and on the track they are following. Some suggestions for individual tracks are indicated by plus signs in Tables 1–4. A plus to the right of a course symbol (whether prerequisite or core) indicates that advanced work in the topic area of that course is recommended for students in that track. Often some specific suggestions for additional study are indicated in the “Going Further” sections of the course catalog, but where specialized courses are not to be found online (as is likely), one hopes that the basic course has provided sufficient background for the student to learn by self-study of more advanced texts and journals.

For Bioinformatics Analysis, additional biology coursework or other study would be required for the student to approach problems with the expected degree of domain sophistication, so that interpretations of data are placed in an appropriate biological context. Ideally this would include exposure to laboratory science, which of course is unlikely in the case of online learners. However, it is expected that many individuals embarking on this track would already be degreed biologists who are seeking additional training to do advanced analyses with their own data or that of others. To some degree the same may be true of the Data Mining track, though these individuals are probably more likely to be committed to a career in exclusively “dry” biology.

Students in the two software tracks, Bioinformatics Tools and Bioinformatics Systems, may wish to take additional courses in subjects such as machine architecture, operating systems, or theory of programming languages, but by far the most important requirement for independent study is actual programming experience. These individuals would be well advised to take on substantial projects in the biological domain that go beyond the requirements of the courses taken.

Finally, the Computational Biology track may call for independent study in a variety of topics in advanced mathematics and computer science as well as biological background necessary for a particular specialization. The curriculum offered here is slanted toward systems biology in this regard, but individuals may prefer to study topics such as evolutionary dynamics or mathematical genetics that would require additional study.

## Conclusion

As noted at the outset, any proposed curriculum must be based on the shifting sands of available offerings, and moreover is necessarily a matter of opinion, both scientific and pedagogical. Without a doubt there are gaps, and quality is not uniform. For instance, there are few suitable resources in important areas such as neuroscience and structural biology, and several other areas are thin. But the offerings are only getting better and more numerous, and so any imperfections in the current collection should be increasingly easy to correct with the passage of time. A more pertinent question is whether an online education is an adequate substitute for what is termed a resident education, in general and in the particular case of bioinformatics.

One undeniable truism is that independent study requires motivation and discipline in the extreme. Students must be committed to doing assigned readings, exercises and assessments faithfully to achieve maximum uptake, the more so for being on their own. A companion article by the author, “Ten Simple Rules for Online Learning” [44], attempts to provide practical advice along these lines.

A particular piece of advice it offers is to pay special attention to doing programming projects in the biological domain. One great risk to the proposition of online bioinformatics education is that students never really get to grips with applying newfound computational or analytic skills to real biological data and actual problems in the full context of the scientific establishment. To be sure, biological databases are readily accessible and datasets may be found online that can serve as challenge problems for classification, and so forth. But that is not the same as the interactive process of designing a novel experimental program, acquiring data direct from instrumentation, cleaning and reducing it, and taking responsibility for storing it in both persistent and queryable form. Nor does classroom learning by itself, virtual or otherwise, fully prepare one for establishing real-world error models, dealing with missing data, establishing a statistical case for some result, arguing and defending scientific positions, navigating the publication process, and sundry other practical skills.

Thus, a useful adjunct to online learning in bioinformatics might be a portfolio of suggested projects based on real-world datasets that would help exercise the skills of trainees, perhaps in the context of an online community of peers. One can even imagine a future in which the use of virtual laboratories makes it possible for students to undertake mixed wet/dry studies of their own. Just as the Amazon Cloud now makes large-scale computing accessible and economically feasible without the support of a large institutional data center, the decreasing cost of sequencing technology and the synthetic biology movement are both suggestive of the possibility of analogous sorts of remote biology. Educational grants for the creation of virtual laboratories to enrich the online learning experience might be public (or philanthropic) money well spent.

Any amount of study in any context cannot substitute for immersion in the social context of science. In an online learning environment, direct interaction with peers is certainly possible after a fashion, through discussion logs and the like, but to date hasn't addressed such important educational elements as the development of public speaking skills. Perhaps the last great barrier to self-learning is the absence of an advisor, with all that implies, and of membership in a working lab. Even the most imaginative web technology will only go so far in this regard, and probably not far enough in the case of wet biology. However, the field of bioinformatics by its nature may offer the best chance for finding ways to involve distance learners directly in ongoing scientific research, and that would seem to be a worthy goal for the burgeoning online education movement.
